# Supplementary material for: Soil carbon debt from land use change in Brazil
Source: Nat Commun. 2026 Jan 26;17:1626. doi: 10.1038/s41467-026-68340-4 (PMC12905268; doi:10.1038/s41467-026-68340-4)
Supplement: Supplementary file 1 — Supplementary Information [file 41467_2026_68340_MOESM1_ESM.pdf]

-Supplementary Information-

Soil carbon debt from land use change in Brazil

João M. Villela[1], Júnior M. Damian[2], Daniel R. P. Gonçalves[3], Luis G. Barioni[2], Maurício R. Cherubin[1,4] & Carlos E. P. Cerri[1,4]*

[1]Department of Soil Science, Luiz de Queiroz College of Agriculture, University of São Paulo, Av. Pádua Dias, 11, Piracicaba, São Paulo, 13418-260, Brazil.

[2]Embrapa Digital Agriculture, Av. Dr. André Tosello, 209, Campinas, São Paulo,13083-886, Brazil.

[3]Graduate Program in Agronomy, State University of Ponta Grossa, Av. General Carlos Cavalcanti, 4748, Ponta Grossa, Paraná, 84030-900, Brazil.

[4]Center for Carbon Research in Tropical Agriculture (CCARBON), University of São Paulo, Avenida Pádua Dias, 11, Piracicaba, São Paulo, 13418-900, Brazil.

*Corresponding author: cepcerri@usp.br

Table of content

S1. Supplementary Tables --3

S2. Supplementary Figures --35

S3. Supplementary Discussion ---44

S4. Supplementary References---45

S5. Supplementary Database References--46

S1. Supplementary Tables

Table S1 Average, standard deviation (S.D.), and number of observations (n) of soil organic carbon stocks (Mg C ha^{-1}) for native vegetation (NV) and agriculture (AGR)in the four evaluated soil layers of the Brazilian biomes.

							Average (Mg C ha^{-1})							
**Land Use**	**Soil layer (cm)**	**n**	**Amazon**	**n**	**Caatinga**	**n**	**Cerrado**	**n**	**Atlantic Forest**	**n**	**Pampa**	**n**	**Pantanal**
**NV**	0 - 10	18	23.7 ± 6.2	31	18.9± 7.8	85	25.8 ± 9.6	52	35.1 ± 16.4	14	30.1 ± 6.4	4	17.0 ± 4.0
	0 - 20	18	39.4 ± 10.1	27	31.0 ± 13.3	110	49.7 ± 19.5	75	64.2 ± 22.7	21	45.1 ± 12.7	7	32.3 ± 10.0
	0 - 30	79	46.5 ± 13.1	24	43.9 ± 20.9	79	58.7 ± 19.7	48	75.7 ± 29.9	4	55.5 ± 20.2	5	35.0 ± 15.2
	0 - 100	21	82.9 ± 14.2	8	87.0 ± 29.6	24	136.3 ± 50.2	22	156.1 ± 38.9	-	-	-	-
**AGR**	0 - 10	97	22.7 ± 6.8	74	15.1 ± 7.8	412	22.5 ± 7.7	406	24.5 ± 11.7	56	22.0 ± 9.9	7	11.6 ± 5.5
	0 - 20	86	37.6 ± 11.0	94	22.4 ± 10.9	446	41.9 ± 14.1	384	47.1 ± 18.2	121	35.8 ± 11.1	6	16.2 ± 6.0
	0 - 30	183	48.5 ± 13.3	54	33.2 ± 14.7	323	57.4 ± 18.3	334	63.2 ± 22.7	54	50.4 ± 10.4	7	30.3 ± 11.3
	0 - 100	23	84.4 ± 23.7	10	82.0 ± 18.1	131	125.5 ± 43.6	186	134.0 ± 47.3	20	132.8 ± 16.7	-	-

Table S2 Comparative statistical analysis between native vegetation soil organic carbon stocks averages in soil layers of the Brazilian biomes.

Soil layer (cm)	Biomes	Amazon	Caatinga	Cerrado	Atlantic Forest	Pampa	Pantanal
10*	**Amazon**	-					
	**Caatinga**	0.996	-				
	**Cerrado**	1.000	0.012	-			
	**Atlantic Forest**	0.105	<0.001	0.023	-		
	**Pampa**	0.751	0.002	1.000	1.000	-	
	**Pantanal**	1.000	1.000	1.000	0.156	1.000	-
20**	**Amazon**	-					
	**Caatinga**	1.000	-				
	**Cerrado**	0.546	<0.001	-			
	**Atlantic Forest**	0.001	<0.001	0.011	-		
	**Pampa**	1.000	0.129	1.000	0.004	-	
	**Pantanal**	1.000	1.000	0.004	<0.001	1.000	-
30**	**Amazon**	-					
	**Caatinga**	1.000	-				
	**Cerrado**	0.004	0.011	-			
	**Atlantic Forest**	<0.001	<0.001	0.005	-		
	**Pampa**	1.000	1.000	1.000	1.000	-	
	**Pantanal**	1.000	1.000	0.013	0.006	1.000	-
100**	**Amazon**	-					
	**Caatinga**	1.000	-				
	**Cerrado**	1.000	0.001	-			
	**Atlantic Forest**	<0.001	0.001	0.140	-		
	**Pampa**	-	-	-	-	-	
	**Pantanal**	-	-	-	-	-	-

* Shapiro-Wilk test: normal distribution (Tukey HSD - ANOVA);
** Shapiro-Wilk test: non-normal distribution (Dunn's multiple-comparison test results for Kruskal - Wallis analysis of variance on ranks (Adjust method: Bonferroni));
Red color font indicates a significant difference ($P < 0.05$)

Table S3 Comparative statistical analysis between agriculture soil organic carbon stocks averages in soil layers of the Brazilian biomes.

Soil layer (cm)	Biome	Amazon	Caatinga	Cerrado	Atlantic Forest	Pampa	Pantanal
10*	**Amazon**	-					
	**Caatinga**	<0.001	-				
	**Cerrado**	1.000	<0.001	-			
	**Atlantic Forest**	1.000	<0.001	0.417	-		
	**Pampa**	1.000	0.007	1.000	0.160	-	
	**Pantanal**	0.014	1.000	0.008	0.002	0.005	-
20**	**Amazon**	-					
	**Caatinga**	<0.001	-				
	**Cerrado**	0.013	<0.001	-			
	**Atlantic Forest**	<0.001	<0.001	<0.001	-		
	**Pampa**	1.000	<0.001	<0.001	<0.001	-	
	**Pantanal**	0.006	1.000	0.002	<0.001	0.007	-
30*	**Amazon**	-					
	**Caatinga**	<0.001	-				
	**Cerrado**	<0.001	<0.001	-			
	**Atlantic Forest**	<0.001	<0.001	0.004	-		
	**Pampa**	1.000	<0.001	0.169	<0.001	-	
	**Pantanal**	0.267	1.000	0.004	<0.001	0.173	-
100**	**Amazon**	-					
	**Caatinga**	1.000	-				
	**Cerrado**	0.001	0.005	-			
	**Atlantic Forest**	<0.001	<0.001	0.524	-		
	**Pampa**	-	-	-	-	-	
	**Pantanal**	-	-	-	-	-	-

*Shapiro-Wilk test: normal distribution (Tukey HSD - ANOVA);
**Shapiro-Wilk test: non-normal distribution (Dunn's multiple-comparison test results for Kruskal - Wallis analysis of variance on ranks (Adjust method: Bonferroni));
Red color font indicates a significant difference ($P < 0.05$)

Table S4 Accumulated annual precipitation (mm), average temperature yearly (°C), and types of climates (Köppen's climate classification) present in Brazilian biomes.

**Biome**	**Tropical zone**				**Arid**	**Temperate zone**				**Annual average temperature (°C)**	**Annual accumulated rainfall (mm)**
	**Af**	**Am**	**Aw**	**As**	**BSh**	**Cfa**	**Cfb**	**Cwa**	**Cwb**		
	**%**										
**Overall**	22.6	27.5	25.8	5.5	4.9	6.5	2.6	2.5	2.1	-	-
**Atlantic Forest**	5.2	3.6	20.1	5.4	0.1	28.6	18.8	9	9.2	26.2 ± 1.0	2,434 ± 344
**Cerrado**	0.8	9	75	3.8	-	2.2	0.2	5.6	3.3	24.5 ± 1.5	877 ± 353
**Amazon**	43.2	50	6.3	0.4	-	0.1	-	0.1	-	22.3 ± 1.7	1,639 ± 312
**Caatinga**	-	-	13	37.3	48	-	0.4	0.1	1	20.0 ± 3.0	1,600 ± 284
**Pantanal**	4.6	22.1	73.2	-	-	-	-	-	-	18.0 ± 0.7	1,651 ± 155
**Pampa**	-	-	-	-	-	98.7	1.3	-	-	25.1 ± 0.3	1,461 ± 77

Köppen's climate classification - **Af:** Tropical rainforest; **Am:** Tropical monsoon; **As:** Tropical Savannah (winter rain), **Aw:** Tropical Savannah (summer rain); **BSh:** Arid steppe, hot; **Cfa:** Temperate, no dry season, hot summer; **Cfb:** Temperate, no dry season, warm summer; **Cwa:** Temperate, dry winter, hot summer; **Cwb:** Temperate, dry winter, warm summer.

Table S5 Comparative statistical analysis between the soil organic carbon stocks average of the native vegetation and agriculture climatic zones in the four soil layers analyzed.

Native Vegetation					Agriculture				
**Soil layer (cm)**	**Climate Zones**	**Subtropical**	**Tropical**	**Arid**	**Soil layer (cm)**	**Climate Zones**	**Subtropical**	**Tropical**	**Arid**
10*	Subtropical	-			10**	Subtropical	-		
	Tropical	< .001	-			Tropical	0.165	-	
	Arid	< .001	0.009	-		Arid	<0.001	<0.001	-
20**	Subtropical	-			20**	Subtropical	-		
	Tropical	0.009	-			Tropical	<0.001	-	
	Arid	< .001	0.006	-		Arid	<0.001	<0.001	-
30*	Subtropical	-			30**	Subtropical	-		
	Tropical	< .001	-			Tropical	<0.001	-	
	Arid	< .001	0.025	-		Arid	<0.001	<0.001	-
100**	Subtropical	-			100**	Subtropical	-		
	Tropical	>0.05	-			Tropical	<0.001	-	
	Arid	>0.05	>0.05	-		Arid	0.05	0.004	-

Köppen's climate classification - **Af:** Tropical rainforest; **Am:** Tropical monsoon; **As:** Tropical Savannah (winter rain), **Aw:** Tropical Savannah (summer rain); **BSh:** Arid steppe, hot; **Cfa:** Temperate, no dry season, hot summer; **Cfb:** Temperate, no dry season, warm summer; **Cwa:** Temperate, dry winter, hot summer; **Cwb:** Temperate, dry winter, warm summer.

*Shapiro-Wilk test: normal distribution (Tukey HSD - ANOVA);

**Shapiro-Wilk test: non-normal distribution (Dunn's multiple-comparison test results for Kruskal - Wallis analysis of variance on ranks (Adjust method: Bonferroni));

Red color font indicates a significant difference (P <0.05)

Table S6 Comparison between the soil organic carbon stocks averages (standard deviation in parentheses) of native vegetation (NV) and agriculture (AGR) in the climate types present in the biomes in the 0 – 10 cm layer.

	**Climate Zones**																	
	**Tropical**								**Arid**		**Subtropical**							
**Biome**	**Af**		**Am**		**As**		**Aw**		**BSh**		**Cfa**		**Cfb**		**Cwa**		**Cwb**	
	NV	AG	NV	AG	NV	AG	NV	AG	NV	AG	NV	AG	NV	AG	NV	AG	NV	AG
**Overall**	30.6 (12.3)	36.2 (11.9)	25.4 (6.5)	23.7 (8.6)	18.2 (8.2)	22.6 (20.5)	26.8 (12.3)	22.0 (8.1)	19.5 (8.4)	15.2 (8.4)	32.9 (12.2)	22.9 (9.0)	37.7 (17.5)	30.4 (10.2)	27.5 (6.9)	19.2 (10.1)	38.5 (4.8)	34.9 (11.7)
**Amazon**			24.8 (5.3)	22.1 (7.0)			18.1											
**Caatinga**					19.2 (7.4)	13.8 (9.0)			18.8 (7.7)	13.7 (5.0)								
**Cerrado**			28.2 (10.3)	25.9 (8.1)	24.4	18.4 (6.0)	24.3 (8.3)	22.2 (6.9)			28.6	21.0 (3.4)			32.9 (12.8)	21.4 (9.9)		
**Atlantic Forest**	31.6 (9.7)	36.2 (10.2)	27.0 (9.3)	21.7 (11.1)	16	10.1	29.1 (11.2)	23.5 (11.7)			26.0 (12.3)	20.1 (10.2)	41.3 (18.0)	30.0 (11.5)			30.8 (5.1)	24.0 (9.8)
**Pampa**											30.0 (6.4)	21.4 (6.5)						
**Pantanal**							16.59 (4.5)	9.7 (3.4)										

Köppen's climate classification - **Af:** Tropical rainforest; **Am:** Tropical monsoon; **As:** Tropical Savannah (winter rain), **Aw:** Tropical Savannah (summer rain); **BSh:** Arid steppe, hot; **Cfa:** Temperate, no dry season, hot summer; **Cfb:** Temperate, no dry season, warm summer; **Cwa:** Temperate, dry winter, hot summer; **Cwb:** Temperate, dry winter, warm summer.

Table S7 Comparison between the soil organic carbon stocks averages (standard deviation in parentheses) of native vegetation (NV) and agriculture (AGR) in the climate types present in the biomes in the 0 – 20 cm layer.

Biome	Climate Zones																	
	Tropical								Arid		Subtropical							
	Af		Am		As		Aw		BSh		Cfa		Cfb		Cwa		Cwb	
	NV	AG	NV	AG	NV	AG	NV	AG	NV	AG	NV	AG	NV	AG	NV	AG	NV	AG
**Overall**	48.0 (14.7)	55.7 (23.6)	43.6 (12.5)	38.4 (12.0)	36.5 (17.4)	24.5 (15.2)	51.3 (22.5)	41.3 (14.0)	30.0 (15.5)	19.9 (9.6)	52.4 (19.3)	40.9 (14.5)	70.9 (21.6)	56.0 (16.8)	51.0 (12.5)	39.4 (12.8)	68.9 (7.0)	58.0 (22.3)
**Amazon**			48.8 (19.5)	38.0 (12.7)														
**Caatinga**					32.1 (10.8)	20.3 (8.9)			30.0 (15.5)	23.3 (8.7)								
**Cerrado**			56.8 (14.3)	50.9 (11.9)	60.9	32.7 (9.8)	48.8 (16)	41.9 (13.1)			46.6	37.0 (5.5)			57.1 (25.0)	39.8 (17.5)		
**Atlantic Forest**	57.9 (17.1)	63.9 (16.2)	52.6 (14.02)	45.1 (11.1)	52.0 (17.4)	45.82 (39.0)	46.9	35.8 (12.8)			60.4 (21.5)	47.8 (15.5)	73.6 (23.3)	55.9 (18.0)	59.9 (15.6)		51.8 (10.6)	37.7 (12.4)
**Pampa**											45.1 (12.7)	37.2 (11.1)						
**Pantanal**							32.3 (9.9)	16.23 (6.0)										

Köppen's climate classification- **Af:** Tropical rainforest; **Am:** Tropical monsoon; **As:** Tropical Savannah (winter rain), **Aw:** Tropical Savannah (summer rain); **BSh:** Arid steppe, hot; **Cfa:** Temperate, no dry season, hot summer; **Cfb:** Temperate, no dry season, warm summer; **Cwa:** Temperate, dry winter, hot summer; **Cwb:** Temperate, dry winter, warm summer.

Table S8 Comparison between the soil organic carbon stocks averages (standard deviation in parentheses) of native vegetation (NV) and agriculture (AGR) in the climate types present in the biomes in the 0 – 30 cm layer.

	Climate Zones																	
	Tropical								Arid		Subtropical							
**Biome**	**Af**		**Am**		**As**		**Aw**		**BSh**		**Cfa**		**Cfb**		**Cwa**		**Cwb**	
	**NV**	**AGR**	**NV**	**AGR**	**NV**	**AGR**	**NV**	**AGR**	**NV**	**AGR**	**NV**	**AGR**	**NV**	**AGR**	**NV**	**AGR**	**NV**	**AGR**
**Overall**	43.0 (18.7)	61.0 (29.8)	47.7 (11.7)	49.9 (14.1)	51.8 (26.6)	46.6 (24.0)	57.0 (21.2)	56.1 (18.3)	40.2 (20.0)	23.5 (12.4)	67.7 (28.5)	58.7 (17.2)	95.2 (32.2)	75.0 (18.6)	65.9 (16.4)	60.5 (22.1)	58.64	60.5 (30.6)
**Amazon**			46.1 (12.3)	46.6 (10.9)				34.8 (5.6)										
**Caatinga**					46.5 (19.7)	34.4 (16.5)			40.4 (22.8)	29.1 (11.0)								
**Cerrado**			34.6 (9.1)	57.4 (34.4)		53.4 (8.6)	58.5 (16.9)	58.7 (16.3)			44.8	50.5 (11.0)			74.2 (24.5)	62.5 (26.50)		
**Atlantic Forest**	73.3 (27.9)	80.9 (27.0)	43.4	58.2 (14.8)	40.3	26.5	39.3	35.5 (15.2)			73.6 (31.4)	63.3 (19.0)	93.2 (27.1)	82.5 (24.7)		68.9 (19.0)	63.8 (14.9)	62.1 (28.9)
**Pampa**											55.5 (20.2)							
**Pantanal**							35.0 (15.1)	30.3 (11.3)										

Köppen's climate classification - **Af:** Tropical rainforest; **Am:** Tropical monsoon; **As:** Tropical Savannah (winter rain), **Aw:** Tropical Savannah (summer rain); **BSh:** Arid steppe, hot; **Cfa:** Temperate, no dry season, hot summer; **Cfb:** Temperate, no dry season, warm summer; **Cwa:** Temperate, dry winter, hot summer; **Cwb:** Temperate, dry winter, warm summer.

Table S9 Comparison between the soil organic carbon stocks averages (standard deviation in parentheses) of native vegetation (NV) and agriculture (AGR) in the climate types present in the biomes in the 0 – 100 cm layer.

Biome	Climate Zones																	
	Tropical								Arid		Subtropical							
	Af		Am		As		Aw		BSh		Cfa		Cfb		Cwa		Cwb	
	NV	AGR	NV	AGR	NV	AGR	NV	AGR	NV	AGR	NV	AGR	NV	AGR	NV	AGR	NV	AGR
**Overall**	92.2 (35.0)	170.7 (34.8)	86.7 (16.1)	113.2 (36.9)	96.9 (45.6)	-	132.6 (49.5)	117.1 (43.9)	106.7 (38.2)	79.4	160.5 (43.4)	127.2 (40.5)	152.5 (42.9)	180.9 (42.6)	157.2 (35.0)	128.5 (36.0)	-	-
**Amazon**			90.3 (18.6)	95.4 (18.8)														
**Caatinga**					74.9 (13.8)	81.5				63.8								
**Cerrado**			54.6	57.6		106. 6 (7.6)	146.0 (40.0)	133.8 (27.1)			75.6	82.3 (1.9)			170.6 (36.5)	136.9 (30.8)		
**Atlantic Forest**	139.2	170.7 (34.7)	83.5	92.1 (5.6)	184.7			86.1 (22.4)			156.4 (42.1)	138.5 (35.2)	168.1 (132.7)	172.7 (34.2)		120.4 (16.5)		
**Pampa**											116.8	135.4 (16.9)						
**Pantanal**																		

Köppen's climate classification - **Af:** Tropical rainforest; **Am:** Tropical monsoon; **As:** Tropical Savannah (winter rain), **Aw:** Tropical Savannah (summer rain); **BSh**: Arid steppe, hot; **Cfa:** Temperate, no dry season, hot summer; **Cfb:** Temperate, no dry season, warm summer; **Cwa:** Temperate, dry winter, hot summer; **Cwb:** Temperate, dry winter, warm summer.

Table S10 Comparative statistical analysis between climate types SOC stocks average at the native vegetation in soil layers of the biomes.

Soil layers (cm)	Climate types	Af	Am	As	Aw	BSh	Cfa	Cfb	Cwa	Cwb
	**Af**	-								
	**Am**	1.000	-							
	**As**	1.000	0.766	-						
	**Aw**	1.000	1.000	0.148	-					
**10****	**BSh**	1.000	0.790	1.000	0.044	-				
	**Cfa**	1.000	1.000	0.002	0.411	<0.001	-			
	**Cfb**	1.000	0.950	<0.001	0.151	<0.001	1.000	-		
	**Cwa**	1.000	1.000	0.222	1.000	0.191	1.000	1.000	-	
	**Cwb**	1.000	1.000	0.081	1.000	0.099	1.000	1.000	1.000	-
	**Af**	-								
	**Am**	1.000	-							
	**As**	1.000	1.000	-						
	**Aw**	1.000	1.000	0.176	-					
**20****	**BSh**	0.727	1.000	1.000	0.022	-				
	**Cfa**	1.000	1.000	0.167	1.000	0.022	-			
	**Cfb**	1.000	0.011	<0.001	0.023	<0.001	0.178	-		
	**Cwa**	1.000	1.000	0.831	1.000	0.152	1.000	0.848	-	
	**Cwb**	1.000	0.982	0.169	1.000	0.049	1.000	1.000	1.000	-
	**Af**	-								
	**Am**	0.934	-							
	**As**	0.979	1.000	-						
**30****	**Aw**	0.223	0.450	0.921	-					
	**BSh**	0.996	0.450	0.702	0.025	-				
	**Cfa**	0.047	0,109	0.432	0.810	0.005	-			
	**Cfb**	<0.001	<0.001	<0.001	<0.001	<0.001	0.106	-		

	**Cwa**	0.026	0.060	0.282	0.598	0.003	1.000	0.287	-	
	**Cwb**	-	-	-	-	-	-	-	-	-
	**Af**	-								
	**Am**	1.000	-							
	**As**	1.000	-	-						
	**Aw**	1.000	1.000	1.000	-					
**100****	**BSh**	1.000	1.000	1.000	1.000	-				
	**Cfa**	0.247	0.498	0.588	1.000	1.000	-			
	**Cfb**	0.388	0.735	0.844	1.000	1.000	1.000	-		
	**Cwa**	0.524	0.916	1.000	1.000	1.000	1.000	1.000	-	
	**Cwb**	-	-	-	-	-	-	-	-	-

Köppen climate classification - **Af:** Tropical rainforest; **Am:** Tropical monsoon; **As:** Tropical Savannah (winter rain), **Aw:** Tropical Savannah (summer rain); **BSh:** Arid steppe, hot; **Cfa:** Temperate, no dry season, hot summer; **Cfb:** Temperate, no dry season, warm summer; **Cwa:** Temperate, dry winter, hot summer; **Cwb:** Temperate, dry winter, warm summer.

*Shapiro-Wilk test: normal distribution (Tukey HSD - ANOVA);

**Shapiro-Wilk test: non-normal distribution (Dunn's multiple-comparison test results for Kruskal - Wallis analysis of variance on ranks (Adjust method: Bonferroni));

Red color font indicates a significant difference (P <0.05)

Table S11 Comparative statistical analysis between climate types SOC stocks average at the agriculture in soil layers of the biomes.

Soil layers (cm)	Climate types	Af	Am	As	Aw	BSh	Cfa	Cfb	Cwa	Cwb
10**	**Af**	-								
	**Am**	0.012	-							
	**As**	<0.001	0.162	-						
	**Aw**	<0.001	1.000	0.499	-					
	**BSh**	<0.001	<0.001	1.000	<0.001	-				
	**Cfa**	<0.001	1.000	0.339	1.000	<0.001	-			
	**Cfb**	1.000	0.010	<0.001	<0.001	<0.001	<0.001	-		
	**Cwa**	<0.001	0.027	1.000	0.025	0.419	0.022	<0.001	-	
	**Cwb**	1.000	0.385	<0.001	0.033	<0.001	0.066	<0.001	<0.001	-
20**	**Af**	-								
	**Am**	0.421	-							
	**As**	<0.001	<0.001	-						
	**Aw**	1.000	1.000	<0.001	-					
	**BSh**	<0.001	<0.001	1.000	<0.001	-				
	**Cfa**	0.834	1.000	<0.001	1.000	<0.001	-			
	**Cfb**	1.000	<0.001	<0.001	<0.001	<0.001	<0.001	-		
	**Cwa**	0.556	1.000	<0.001	1.000	<0.001	1.000	<0.001	-	
	**Cwb**	1.000	0.669	<0.001	1.000	<0.001	1.000	1.000	0.863	-
30**	**Af**	-								
	**Am**	1.000	-							
	**As**	1.000	1.000	-						
	**Aw**	1.000	0.018	0.227	-					
	**BSh**	<0.001	<0.001	0.423	<0.001	-				
	**Cfa**	1.000	0.002	0.051	1.000	<0.001	-			
	**Cfb**	0.072	<0.001	<0.001	<0.001	<0.001	<0.001	-		

	**Cwa**	1.000	0.007	1.000	1.000	<0.001	1.000	<0.001	-	
	**Cwb**	1.000	1.000	1.000	1.000	0.354	1.000	0.787	1.000	-
**100****	**Af**	-								
	**Am**	0.032	-							
	**As**	-	-	-						
	**Aw**	0.033	1.000	-	-					
	**BSh**	0.009	1.000	-	1.000	-				
	**Cfa**	0.329	1.000	-	1.000	0.447	-			
	**Cfb**	1.000	<0.001	-	<0.001	<0.001	<0.001	-		
	**Cwa**	0.509	1.000	-	1.000	0.395	1.000	<0.001	-	
	**Cwb**	-	-	-	1.000	-	-	-	-	-

Köppen climate classification - **Af:** Tropical rainforest; **Am:** Tropical monsoon; **As:** Tropical Savannah (winter rain), **Aw:** Tropical Savannah (summer rain); **BSh:** Arid steppe, hot; **Cfa:** Temperate, no dry season, hot summer; **Cfb:** Temperate, no dry season, warm summer; **Cwa:** Temperate, dry winter, hot summer; **Cwb:** Temperate, dry winter, warm summer.

Table S12 Soil organic carbon stocks average (SD: standard deviation in parentheses) and max and min values in the soil classes present in the biomes to soil layers of native vegetation and agriculture.

**Soil Organic Carbon Stocks (Mg ha^{-1})**												
**Native Vegetation**												
**Layer(cm)**	**0 - 10**			**0 - 20**			**0 - 30**			**0 - 100**		
**Soil class**	**Min**	**Avg. (SD)**	**Max**	**Min**	**Avg. (SD)**	**Max**	**Min**	**Avg. (SD)**	**Max**	**Min**	**Avg. (SD)**	**Max**
**Alfisol**	7.0	20.9 (9.4)	34.1	7	29.1 (14.0)	50.4	18.0	33.5 (17.1)	67.3	-	-	-
**Entisol**	8.3	23.0 (21.9)	95.7	15.0	31.1 (17.0)	73.3	17.4	33.5 (11.8)	159.7	-	-	-
**Inceptisol**	13.0	33.1 (11.7)	66.9	56.3	70.4 (18.2)	98.0	30.8	60.5 (27.3)	119.7	60.3	99.4 (37.2)	165.4
**Ultisol**	8.31	23.1 (9.1)	40.0	14.3	39.47(13.7)	72.7	25.3	46.9(16.4)	98.7	63.5	89.2(30.6)	181.0
**Oxisol**	11.4	28.3(10.7)	66.3	9.3	54.7(20.5)	136.2	21.7	61.1(21.8)	137.3	54.6	138.9(44.7)	224.5
**Agriculture**												
**Layer(cm)**	**0 - 10**			**0 - 20**			**0 - 30**			**0 - 100**		
**Soil Class**	**Min**	**Avg. (SD)**	**Max**	**Min**	**Avg. (SD)**	**Max**	**Min**	**Avg. (SD)**	**Max**	**Min**	**Avg. (SD)**	**Max**
**Alfisol**	7.0	18.1 (7.7)	35.7	7.0	30.2 (13.5)	62.4	9.0	35.6 (17.3)	62.2	84.3	111.4 (23.3)	147.7
**Entisol**	5.4	13.0 (7.25)	43.0	10.0	21.4 (12.3)	83.3	12.1	34.5 (16.3)	79.2	-	-	-
**Inceptisol**	12.9	27.8 (9.4)	50.0	21.3	49.1(17.1)	92.3	38.1	69.0 (15.5)	107.1	48.3	156.7 (72.2)	274.0
**Ultisol**	4.6	20.0 (10.2)	63.0	7.8	33.25(14.1)	90.0	12.0	48.5 (17.2)	120	54.2	125.8 (46.2)	214.0
**Oxisol**	5.1	23.8(9.1)	61.3	10.4	44.2 (15.1)	123.0	19.9	59.5 (18.8)	121.56	27.4	129.9(43.7)	249.5

Table S13 Comparative statistical analysis between soil classes SOC stocks average at the native vegetation and agriculture in soil layers.

	Native Vegetation						Agriculture					
**Soil Layer(cm)**	**Soil Class**	**Alfisol**	**Entisol**	**Inceptisol**	**Ultisol**	**Oxisol**	**Soil Class**	**Alfisol**	**Entisol**	**Inceptisol**	**Ultisol**	**Oxisol**
**0 - 10**	**Alfisol**	-					**Alfisol**	-				
	**Entisol**	1.000	-				**Entisol**	0.305	-			
	**Inceptisol**	0.024	0.003	-			**Inceptisol**	<0.001	<0.001	-		
	**Ultisol**	1.000	1.000	0.018	-		**Ultisol**	1.000	<0.001	<0.001	-	
	**Oxisol**	0.150	0.016	1.000	0.067	-	**Oxisol**	0.022	<0.001	0.078	<0.001	-
**0 - 20**	**Alfisol**	-					**Alfisol**	-				
	**Entisol**	1.000	-				**Entisol**	0.143	-			
	**Inceptisol**	<0.001	<0.001	-			**Inceptisol**	<0.001	<0.001	-		
	**Ultisol**	1.000	1.000	<0.001	-		**Ultisol**	1.000	<0.001	<0.001	-	
	**Oxisol**	<0.001	<0.001	0.137	<0.001	-	**Oxisol**	<0.001	<0.001	0.697	<0.001	-
**0 - 30**	**Alfisol**	-					**Alfisol**	-				
	**Entisol**	0.998	-				**Entisol**	1.000	-			
	**Inceptisol**	0.012	< 0.001	-			**Inceptisol**	<0.001	<0.001	-		
	**Ultisol**	0.109	0.006	0.442	-		**Ultisol**	1.000	<0.001	<0.001	-	
	**Oxisol**	<0.001	< 0.001	0.994	0.002	-	**Oxisol**	0.010	<0.001	0.045	<0.001	-
**0 - 100**	**Alfisol**	-					**Alfisol**	-				
	**Entisol**	-	-				**Entisol**	-	-			
	**Inceptisol**	-	-	-			**Inceptisol**	-	0.003	-		
	**Ultisol**	-	-	1.000	-		**Ultisol**	-	0.095	1.000	-	
	**Oxisol**	-	-	0.025	0.002	-	**Oxisol**	-	<0.001	1.000	1.00	-

Shapiro-Wilk test: non-normal distribution (Dunn's multiple-comparison test results for Kruskal - Wallis analysis of variance on ranks (Adjust method: Bonferroni)); Red color font indicates a significant difference (P <0.05).

Table S14 Comparative statistical analysis between SOC stocks at different altitudes and soil layers for native vegetation and agriculture in the Atlantic Forest biome.

Native Vegetation					Agriculture				
**Soil layer (cm)**	**Altitude (m.a.m.s.l*)**	**0 - 400**	**401 - 800**	**801 - 1200**	**Soil layer (cm)**	**Altitude (m.a.m.s.l*)**	**0 - 400**	**401 - 800**	**801 - 1200**
**10****	0 - 400	-			10**	0 - 400	-		
	401 - 800	0.920	-			401 - 800	<0.001	-	
	801 - 1200	0.013	0.400	-		801 - 1200	<0.001	<0.001	-
**20****	0 - 400	-			20**	0 - 400	-		
	401 - 800	0.227	-			401 - 800	<0.001	-	
	801 - 1200	0.032	0.930	-		801 - 1200	<0.001	<0.001	-
**30****	0 - 400	-			30**	0 - 400	-		
	401 - 800	0.490	-			401 - 800	0.680	-	
	801 - 1200	0.030	0.570	-		801 - 1200	<0.001	<0.001	-
**100****	0 - 400	-			100**	0 - 400	-		
	401 - 800	0.710	-			401 - 800	0.710	-	
	801 - 1200	0.024	0.630	-		801 - 1200	<0.001	0.061	-

*Altitude - m.a.m.s.l: Meters above sea level.

Table S15 Summary of meta-analysis results stratified by biome and soil depth, presenting effect size estimates with confidence intervals (Random and Mixed models), heterogeneity statistics, and the explanatory influence of the moderator's latitude, mean annual temperature, and annual rainfall accumulation.

**Biome**	**Soil layer (cm)**	**Model**	**Term**	**Estimate**	**SE**	**Zval**	**Pval**	**CI.lb**	**CI.ub**	**τ²**	**I²**	**R²**	**Egger test**	**Fail-Safe-N**	**Trim and Fill method**			
															**Estimate**	**CI.lb**	**CI.ub**	**k0**
		**Null**	intrcpt	-4.4	0.3	-13.2	1.2E-39	-5.1	-3.8	51.4	98.0		0.387	1.0E+06	-4.4	-5.1	-3.8	0
		**Mixed**	intrcpt	-4.4	0.3	-13.6	4.9E-42	-5.0	-3.8	47.5	97.8	7.5						
	**0 - 10**		Temp.	1.2	0.5	2.4	0.02	0.2	2.2									
			Rainfall	0.0	0.4	-0.1	0.95	-0.7	0.7									
			Lat	1.0	0.5	1.9	0.06	-0.1	2.0									
		**Null**	intrcpt	-6.9	0.4	-16.4	0.00	-7.7	-6.1	77.6	99.9	0.8	0.062	2.E+06	-4.4	-5.3	-3.5	70
		**Mixed**	intrcpt	-6.9	0.4	-16.4	1.3E-60	-7.7	-6.1	77.0	99.9							
**Overall**	**0 - 20**		Temp.	-0.3	0.5	-0.7	0.47	-1.2	0.6									
			Rainfall	0.4	0.5	0.9	0.37	-0.5	1.4									
			Lat	1.0	0.4	2.2	0.03	0.1	1.8									
		**Null**	intrcpt	-5.2	0.4	-11.6	6.3E-31	-6.0	-4.3	58.4	97.9	2.9	0.059	5.E+05	-5.2	-6.0	-4.3	0
		**Mixed**	intrcpt	-5.1	0.4	-11.7	1.9E-31	-6.0	-4.3	56.7	97.8							
	**0 - 30**		Temp.	-0.7	0.8	-0.8	0.40	-2.3	0.9									
			Rainfall	1.3	0.4	2.9	0.00	0.4	2.2									
			Lat	1.32	0.80	1.66	0.10	-0.24	2.88									

Biome = biome considered; **Soil layer (cm)** = depth of the soil layer evaluated; **Term** = parameter estimated in the model; **Estimate** = point estimate of the effect; **SE** = standard error of the estimate; **Zval** = Z statistic value; **Pval** = p-value associated with the test; **CI.lb / CI.ub** = lower and upper bounds of the 95% confidence interval; **Model** = fitted model type (random- or mixed-effects); **τ^2 (tau²)** = between-study variance (unexplained heterogeneity); **I^2** = proportion of total variability due to heterogeneity; **H^2** = ratio of total to sampling variance (relative heterogeneity); **R^2** (%) = proportion of heterogeneity explained by moderators.

Table S16 Accumulation potential carbon (Pg C) and carbon dioxide equivalent (Pg CO_{2eq}) to 0 – 30 cm soil layer of Brazil and biomes.

Biome	C (Pg*)	SD	CO_{2eq} (Pg*)	SD	Biome (%)
Amazon	-0.19	0.12	0.70	0.44	13.5
Caatinga	-0.14	0.14	0.51	0.50	9.9
Cerrado	-0.53	0.12	1.93	0.44	37.5
Atlantic Forest	-0.48	0.12	1.80	0.45	34.2
Pampa	-0.05	0.03	0.02	0.11	3.9
Pantanal	-0.01	0.007	0.05	0.03	0.9
Overall	-1.40	0.12	5.2	0.44	100.0

*Pg: Petagram.

Table S17 Summary of meta-analysis results stratified by biome and soil depth, presenting effect size estimates with confidence intervals (Random and Mixed models), heterogeneity statistics, and the explanatory influence of the moderator's latitude, mean annual temperature, and annual rainfall accumulation.

		Model estimates and statistical parameters								Heterogeneity analysis			Bias and robustness analyses					
**Biome**	**Soil layer (cm)**	**Model**	**Term**	**Estimate**	**SE**	**Zval**	**Pval**	**CI.lb**	**CI.ub**	**τ^2**	**I^2**	**R^2**	**Egger test**	**Fail-Safe-N**	**Trim and Fill method**			
															**Estimate**	**CI.lb**	**CI.ub**	**k0**
	**0 - 10**	**Null**	intrcpt	-4.1	0.4	-9	6.60E-21	-5.0	-3.3	50.2	97.2	-	0.006	195,162	-4.1	-5.0	-3.3	0
		**Mixed**	intrcpt	-4.1	0.4	-10	8.80E-23	-4.9	-3.3	45.6	96.9	9.2						
			Temp.	0.0	0.5	0	0.960	-1.0	1.1									
			Rainfall	0.7	0.5	1	0.141	-0.2	1.6									
			Lat	2.5	0.6	4	1.06E-05	1.4	3.6									
**Cerrado**	**0 - 20**	**Null**	intrcpt	-4.9	0.9	-5	1.94E-07	-6.7	-3.1	68.8	96.8	-	0.08	287,950	-5.0	-6.3	-3.8	29
		**Mixed**	intrcpt	-7.2	0.6	-12	1.55E-34	-8.3	-6.0	67.0	96.7	2.5						
			Temp.	-0.7	0.8	-1	0.39	-2.1	0.8									
			Rainfall	-0.3	0.8	0	0.70	-1.8	1.2									
			Lat	1.4	0.6	2	0.02	0.2	2.6									
	**0 - 30**	**Null**	intrcpt	-5.7	0.7	-9	0.00	-6.9	-4.4	59.4	98.3		0.51	159,994	-3.0	-4.9	-1.0	0
		**Mixed**	intrcpt	-5.7	0.7	-9	0.00	-6.9	-4.4	59.3	98.3	0.15						
			Temp.	0.0	1.1	0	0.97	-2.1	2.1									
			Rainfall	1.0	0.7	2	0.13	-0.3	2.3									
			Lat	0.7	1.1	1	0.50	-1.4	2.8									

Biome = biome considered; **Soil layer (cm)** = depth of the soil layer evaluated; **Term** = parameter estimated in the model; **Estimate** = point estimate of the effect (SOC stock gap (Mg ha-1)); **SE** = standard error of the estimate; **Zval** = Z statistic value; **Pval** = p-value associated with the test; **CI.lb / CI.ub** = lower and upper bounds of the 95% confidence interval; **Model** = fitted model type (random- or mixed-effects); **τ^2 (tau²)** = between-study variance (unexplained heterogeneity); **I^2** = proportion of total variability due to heterogeneity; **H^2** = ratio of total to sampling variance (relative heterogeneity); **R^2 (%)** = proportion of heterogeneity explained by moderators.

Table S18 Summary of meta-analysis results stratified by biome and soil depth, presenting effect size estimates with confidence intervals (Random and Mixed models), heterogeneity statistics, and the explanatory influence of the moderator's latitude, mean annual temperature, and annual rainfall accumulation.

	Model estimates and statistical parameters									Heterogeneity analysis			Bias and robustness analyses					
**Biome**	**Soil layer (cm)**	**Model**	**Term**	**Estimate**	**SE**	**Zval**	**Pval**	**CI.lb**	**CI.ub**	**τ²**	**I²**	**R²**	**Egger test**	**Fail-Safe-N**	**Trim and Fill method**			
															**Estimate**	**CI.lb**	**CI.ub**	**k0**
**Atlantic Forest**	**0 - 10**	**Null**	intrcpt	-6.2	0.9	-7	1.02E-12	-7.9	-4.5	74.1	99.2		0.99	154,482	-6.2	-7.9	-4.5	0
		**Mixed**	intrcpt	-6.2	0.7	-9	1.75E-18	-7.5	-4.8	48.0	98.8	35.2						
			Temp.	7.1	1.1	6	9.13E-11	5.0	9.2									
			Rainfall	2.2	0.8	3	8.78E-03	0.6	3.9									
			Lat_z	-1.8	1.1	-2	1.08E-01	-4.0	0.4									
	**0 - 20**	**Null**	intrcpt	-7.7	1.4	-6	0.00	-10.3	-5.0	117.7	97.2		0.055	93,896	-11.6	-14.5	-8.7	16
		**Mixed**	intrcpt	-7.6	1.4	-6	2.57E-08	-10.3	-4.9	116.4	97.0	1.1						
			Temp.	3.3	2.5	1	1.78E-01	-1.5	8.2									
			Rainfall	0.8	1.6	0	6.23E-01	-2.3	3.9									
			Lat	0.0	2.4	0	9.95E-01	-4.6	4.6									
	**0 - 30**	**Null**	intrcpt	-6.7	0.9	-8	4.40E-14	-8.4	-4.9	38.7	94.9		0.26	22,216	-6.7	-8.4	-4.9	0
		**Mixed**	intrcpt	-6.7	0.9	-8	0.00	-8.4	-5.0	37.3	94.5	3.7						
			Temp	2.8	1.5	2	0.06	-0.1	5.7									
			Rainfall	1.9	1.2	2	0.12	-0.5	4.3									
			Lat	-1.4	1.4	-1	0.33	-4.2	1.4									

Biome = biome considered; **Soil layer (cm)** = depth of the soil layer evaluated; **Term** = parameter estimated in the model; **Estimate** = point estimate of the effect; **SE** = standard error of the estimate; **Zval** = Z statistic value; **Pval** = p-value associated with the test; **CI.lb / CI.ub** = lower and upper bounds of the 95% confidence interval; **Model** = fitted model type (random- or mixed-effects); **τ^2 (tau²)** = between-study variance (unexplained heterogeneity); **I^2** = proportion of total variability due to heterogeneity; **H^2** = ratio of total to sampling variance (relative heterogeneity); **R^2 (%)** = proportion of heterogeneity explained by moderators.

Table S19 Summary of meta-analysis results stratified by biome and soil depth, presenting effect size estimates with confidence intervals (Random and Mixed models), heterogeneity statistics, and the explanatory influence of the moderator's latitude, mean annual temperature, and annual rainfall accumulation.

Biome	Soil layer (cm)	Model	Term	Estimate	SE	Zval	Pval	CI.lb	CI.ub	τ^2	I^2	R^2	Egger test	Fail-Safe-N	Trim and Fill method			
															Estimate	CI.lb	CI.ub	k0
Amazon	0 - 10	**Null**	intrcpt	-3.8	0.7	-5.3	1.1E-07	-5.2	-2.4	7.9	77.7	-	0.775	1,132	-3.8	-5.2	-2.4	0
		**Mixed**	intrcpt	-3.7	0.7	-5.0	5.2E-07	-5.1	-2.2	8.1	78.2	-3.4						
			Temp.	-1.0	0.8	-1.3	2.0E-01	-2.5	0.5									
			Rainfall	0.7	0.8	0.9	3.8E-01	-0.9	2.3									
			Lat	-0.2	0.8	-0.2	8.3E-01	-1.8	1.4									
	0 - 20	**Null**	intrcpt	-4.9	0.9	-5.2	1.9E-07	-6.7	-3.1	24.3	97.1	-	0.757	3,351	-3.7	-5.1	-2.3	0
		**Mixed**	intrcpt	-4.9	0.9	-5.6	2.3E-08	-6.6	-3.1	20.1	89.4	17.3						
			Temp.	-2.0	0.9	-2.1	3.2E-02	-3.8	-0.2									
			Rainfall	1.2	0.9	1.3	1.9E-01	-0.6	3.0									
			Lat	-1.8	0.9	-1.9	5.7E-02	-3.6	0.1									
	0 - 30	**Null**	intrcpt	-3.0	1.0	-3.0	2.6E-03	-4.9	-1.0	63.2	97.9		0.100	9,152	-3.0	-4.9	-1.0	0
		**Mixed**	intrcpt	-2.8	0.9	-3.1	1.8E-03	-4.6	-1.1	51.7	96.2	18.3						
			Temp.	-3.2	1.1	-3.0	2.5E-03	-5.3	-1.1									
			Rainfall	2.5	0.9	2.8	5.1E-03	0.8	4.3									
			Lat	1.2	1.0	1.3	2.0E-01	-0.6	3.1									

Biome = biome considered; **Soil layer (cm)** = depth of the soil layer evaluated; **Term** = parameter estimated in the model; **Estimate** = point estimate of the effect; **SE** = standard error of the estimate; **Zval** = Z statistic value; **Pval** = p-value associated with the test; **CI.lb / CI.ub** = lower and upper bounds of the 95% confidence interval; **Model** = fitted model type (random- or mixed-effects); **τ^2 (tau²)** = between-study variance (unexplained heterogeneity); **I^2** = proportion of total variability due to heterogeneity; **H^2** = ratio of total to sampling variance (relative heterogeneity); **R^2 (%)** = proportion of heterogeneity explained by moderators.

Table S20 Summary of meta-analysis results stratified by biome and soil depth, presenting effect size estimates with confidence intervals (Random and Mixed models), heterogeneity statistics, and the explanatory influence of the moderator's latitude, mean annual temperature, and annual rainfall accumulation.

Biome	Soil layer (cm)	Model	Term	Estimate	SE	Zval	Pval	CI.lb	CI.ub	τ^2	I^2	R^2	Egger test	Fail-Safe-N	Trim and Fill method			
															**Estimate**	**CI.lb**	**CI.ub**	**k0**
		**Null**	intrcpt	-2.2	1.1	-2.1	3.5E-02	-4.3	-0.1	46.7	96.9		0.705	1,813	-2.2	-4.3	-0.1	0
		**Mixed**	intrcpt	-2.2	1.1	-2.1	3.5E-02	-4.3	-0.1	45.8	96.7	1.9						
	**0 - 10**		Temp.	1.4	1.4	1.1	2.9E-01	-1.2	4.1									
			Rainfall	-1.7	1.1	-1.6	1.1E-01	-3.7	0.4									
			Lat	-1.1	1.3	-0.9	3.9E-01	-3.8	1.5									
		**Null**	intrcpt	-5.5	1.9	-2.9	4.1E-03	-9.2	-1.7	177.4	99.2		0.175	8,493	-5.5	-9.2	-1.7	0
		**Mixed**	intrcpt	-5.5	1.9	-2.8	4.5E-03	-9.2	-1.7	182.2	99.2	-2.7						
**Caatinga**	**0 - 20**		Temp.	-0.9	2.4	-0.4	7.0E-01	-5.6	3.7									
			Rainfall	-0.8	1.9	-0.4	6.7E-01	-4.6	3.0									
			Lat	-1.8	2.4	-0.8	4.5E-01	-6.4	2.8									
		**Null**	intrcpt	-4.2	2.0	-2.1	3.2E-02	-8.1	-0.4	78.9	98.3		0.403	1,472	-1.8	-6.1	2.5	3
		**Mixed**	intrcpt	-4.2	2.1	-2.0	4.4E-02	-8.3	-0.1	90.0	98.4	-14.0						
	**0 - 30**		Temp.	-0.1	3.1	0.0	9.9E-01	-6.1	6.0									
			Rainfall	-0.6	2.2	-0.3	7.9E-01	-4.9	3.8									
			Lat_z	-1.7	3.1	-0.6	5.8E-01	-7.9	4.4									

Biome = biome considered; **Soil layer (cm)** = depth of the soil layer evaluated; **Term** = parameter estimated in the model; **Estimate** = point estimate of the effect; **SE** = standard error of the estimate; **Zval** = Z statistic value; **Pval** = p-value associated with the test; **CI.lb / CI.ub** = lower and upper bounds of the 95% confidence interval; **Model** = fitted model type (random- or mixed-effects); **τ^2 (tau^2)** = between-study variance (unexplained heterogeneity); **I^2** = proportion of total variability due to heterogeneity; **H^2** = ratio of total to sampling variance (relative heterogeneity); **R^2 (%)** = proportion of heterogeneity explained by moderators.

Table S21 Summary of meta-analysis results stratified by biome and soil depth, presenting effect size estimates with confidence intervals (Random and Mixed models), heterogeneity statistics, and the explanatory influence of the moderator's latitude, mean annual temperature, and annual rainfall accumulation.

Biome	Soil layer (cm)	Model	Term	Estimate	SE	Zval	Pval	CI.lb	CI.ub	τ²	I²	R²	Egger	Fail-Safe-N	Trim and Fill method			
															Estimate	CI.lb	CI.ub	k0
		Null	intrcpt	-5.4	0.8	-6.5	1.1E-10	-7.0	-3.7	16.4	92.0		0.121	5,260	-6.1	-7.8	-4.4	3
		Mixed	intrcpt	-5.3	0.8	-6.8	1.2E-11	-6.9	-3.8	14.3	90.9	12.7						
	0 - 10		Temp.	-1.8	1.1	-1.6	0.11	-4.0	0.4									
			Rainfall	0.9	1.0	1.0	0.34	-1.0	2.8									
			Lat	1.4	1.0	1.4	0.16	-0.5	3.3									
		Null	intrcpt	-7.4	1.0	-7.1	1.2E-12	-9.4	-5.3	77.5	100.0		0.39	72,325	-5.5	-7.7	-3.4	9
		Mixed	intrcpt	-7.4	0.9	-7.8	4.4E-15	-9.2	-5.5	62.7	100.0	19.1						
Pampa	0 - 20		Temp.	-1.3	1.1	-1.2	0.24	-3.6	0.9									
			Rainfall	3.7	1.4	2.6	9.2E-03	0.9	6.6									
			Lat	0.3	1.5	0.2	0.82	-2.7	3.4									
		Null	intrcpt	-6.1	1.9	-3.3	1.1E-03	-9.8	-2.4	78.9	98.1		0.91	1,192	-6.1	-9.8	-2.4	0
		Mixed	intrcpt	-6.3	1.7	-3.7	2.0E-04	-9.6	-3.0	63.6	97.7	19.3						
	0 - 30		Temp.	5.8	5.7	1.0	0.31	-5.3	17.0									
			Rainfall	-5.7	4.8	-1.2	0.23	-15.1	3.7									
			Lat	1.2	3.5	0.3	0.74	-5.7	8.0									

Biome = biome considered; **Soil layer (cm)** = depth of the soil layer evaluated; **Term** = parameter estimated in the model; **Estimate** = point estimate of the effect; **SE** = standard error of the estimate; **Zval** = Z statistic value; **Pval** = p-value associated with the test; **CI.lb / CI.ub** = lower and upper bounds of the 95% confidence interval; **Model** = fitted model type (random- or mixed-effects); **τ² (tau²)** = between-study variance (unexplained heterogeneity); **I²** = proportion of total variability due to heterogeneity; **H²** = ratio of total to sampling variance (relative heterogeneity); **R² (%)** = proportion of heterogeneity explained by moderators.

Table S22 Summary of meta-analysis results stratified by biome and soil depth, presenting effect size estimates with confidence intervals (Random and Mixed models), heterogeneity statistics, and the explanatory influence of the moderator's latitude, mean annual temperature, and annual rainfall accumulation.

Biome	Soil layer (cm)	Model	Term	Estimate	SE	Zval	Pval	CI.lb	CI.ub	τ^2	I^2	R^2	Egger	Fail-Safe-N	Trim and Fill method			
															Estimate	CI.lb	CI.ub	k0
		**Null**	intrcpt	-3.9	0.9	-4.2	3.1E-05	-5.8	-2.1	4.72	90.4		0.223	359	-3.9	-5.8	-2.1	0
		**Mixed**	intrcpt	-4.0	0.8	-4.8	1.9E-06	-5.7	-2.4	3.6	90.5	23.5						
	**0 - 10**		Temp.	0.5	1.2	0.4	0.67	-1.9	2.9									
			Lat	-2.0	1.2	-1.7	0.09	-4.3	0.3									
			-	-	-	-	-	-	-									
		**Null**	intrcpt	-4.8	1.8	-2.7	0.01	-8.2	-1.3	17.7	96.2		0.130	337	-3.8	-7.3	-0.3	1
		**Mixed**	intrcpt	-4.9	1.6	-3.1	1.9E-03	-7.9	-1.8	13.8	96.2	22						
**Pantanal**	**0 - 20**		Temp.	-1.0	2.2	-0.5	0.65	-5.4	3.3									
			Lat	-2.5	2.1	-1.2	0.24	-6.7	1.7									
			-	-	-	-	-	-	-									
		**Null**	intrcpt	-5.3	1.5	-3.4	5.7E-04	-8.3	-2.3	11.2	94.3		0.1		-5.3	-8.3	-2.3	0
		**Mixed**	intrcpt	-5.3	1.4	-3.7	2.1E-04	-8.1	-2.5	9.61	93.2	14						
	**0 - 30**		Temp.	-2.4	1.7	-1.4	0.16	-5.6	0.9									
			Lat.	-1.9	1.7	-1.1	0.25	-5.2	1.4									
			-	-	-	-	-	-	-									

Biome = biome considered; **Soil layer (cm)** = depth of the soil layer evaluated; **Term** = parameter estimated in the model; **Estimate** = point estimate of the effect; **SE** = standard error of the estimate; **Zval** = Z statistic value; **Pval** = p-value associated with the test; **CI.lb / CI.ub** = lower and upper bounds of the 95% confidence interval; **Model** = fitted model type (random- or mixed-effects); **τ^2 (tau²)** = between-study variance (unexplained heterogeneity); **I^2** = proportion of total variability due to heterogeneity; **H^2** = ratio of total to sampling variance (relative heterogeneity); **R^2 (%)** = proportion of heterogeneity explained by moderators.

Table S23 Relative change (%) in soil organic carbon stocks (SOCstocks) across tropical, subtropical, and arid climate classes, stratified by biome.

Soil Layer (cm)	Climate Class	Biome	Pval	Relative change in SOC stocks (%)	CI.lb	CI.ub
0 - 10	Tropical	Cerrado	3.1E-06	-14.1	-19.4	-8.5
		Atlantic Forest	1.9E-02	-19.4	-32.7	-3.4
		Amazon	2.6E-06	-11.7	-16.2	-7.0
		Caatinga	2.4E-02	-18.5	-31.7	-2.7
		Pantanal	7.7E-49	-30.0	-33.3	-26.6
	Subtropical	Atlantic Forest	3.0E-05	-22.7	-31.5	-12.8
		Pampa	1.0E-07	-22.3	-29.2	-14.7
		Cerrado	3.1E-01	-8.5	-22.9	8.6
	Arid	Caatinga	3.1E-01	-10.2	-27.1	10.5
0 - 20	Tropical	Atlantic Forest	9.6E-06	-16.4	-22.8	-9.5
		Amazon	3.0E-07	-11.6	-15.7	-7.4
		Cerrado	2.1E-06	-12.6	-17.4	-7.6
		Caatinga	2.9E-01	-11.3	-29.2	11.0
		Pantanal	2.8E-46	-23.4	-26.2	-20.5
	Subtropical	Atlantic Forest	2.1E-05	-16.00	-22.48	-8.97
		Pampa	6.6E-03	-11.80	-19.45	-3.44
		Cerrado	1.5E-01	-11.26	-24.59	4.44
	Arid	Caatinga	1.0E-01	-13.4	-36.1	4.2
0 - 30	Tropical	Amazon	2.3E-03	-8.0	-12.8	-2.9
		Caatinga	4.9E-01	-7.5	-26.0	15.5
		Cerrado	7.4E-08	-9.7	-12.9	-6.3
		Atlantic Forest	2.3E-03	-16.3	-25.3	-6.2
		Pantanal	3.3E-51	-21.7	-24.2	-19.2
	Subtropical	Cerrado	9.6E-01	-0.4	-15.8	17.7
		Atlantic Forest	7.2E-04	-13.3	-20.2	-5.8
		Pampa	9.3E-01	0.9	-16.9	22.4
	Arid	Caatinga	2.4E-01	-11.5	-27.7	8.4

Table S24 Relative change (%) in soil organic carbon stocks (SOCstocks) by climate class (Tropical: Af, Am, As, Aw; Arid: Bsh; Subtropical: Cfa, Cfb, Cwa, Cwb), stratified by biome.

Soil Layer (cm)	Climate Class	Biome	Pval	Relative change in SOC stocks (%)	CI.lb	CI.ub
0 - 10 cm	Af	Cerrado	3.09E-03	9.2	3.0	15.7
		Atlantic Forest	6.76E-01	-1.2	-6.6	4.5
	Am	Amazon	1.53E-12	-13.6	-17.1	-10.0
		Cerrado	6.73E-02	-11.7	-22.7	0.9
	As	Caatinga	4.55E-03	-23.1	-35.9	-7.8
		Cerrado	2.75E-15	26.4	19.2	33.9
		Atlantic Forest	4.85E-01	-18.7	-54.6	45.5
	Aw	Amazon	3.99E-02	-9.5	-17.8	-0.5
		Caatinga	9.13E-01	-0.9	-15.0	15.7
		Cerrado	1.70E-06	-15.2	-20.7	-9.3
		Atlantic Forest	1.64E-02	-26.0	-42.1	-5.4
		Pantanal	7.74E-49	-22.0	-26.2	-18.5
	Bsh	Caatinga	3.09E-01	-10.2	-27.1	10.5
	Cfa	Atlantic Forest	9.21E-05	-29.0	-40.2	-15.7
		Pampa	1.03E-07	-22.3	-29.2	-14.7
	Cfb	Atlantic Forest	5.72E-06	-16.1	-22.2	-9.5
	Cwa	Cerrado	3.17E-01	-12.3	-32.3	13.5
		Atlantic Forest	2.87E-02	-18.4	-32.0	-2.1
	Cwb	Cerrado	8.66E-01	-0.3	-3.9	3.4
		Atlantic Forest	1.76E-06	12.7	7.3	18.3
0 - 20 cm	Af	Atlantic Forest	2.81E-04	-6.6	-10.0	-3.1
	Am	Amazon	1.47E-02	-10.5	-18.2	-2.2
		Cerrado	5.22E-02	-25.7	-44.9	0.3
	As	Caatinga	2.94E-01	-11.3	-29.2	11.0
		Atlantic Forest	2.28E-01	-17.3	-39.3	12.7
	Aw	Amazon	1.11E-04	-12.5	-18.2	-6.4
		Cerrado	8.39E-06	-12.1	-17.0	-7.0
		Atlantic Forest	1.40E-14	-18.1	-22.2	-13.8

		Pantanal	2.78E-46	-23.4	-26.2	-20.5
	**Bsh**	Caatinga	1.03E-01	-18.4	-36.1	4.2
		Atlantic Forest	4.69E-04	-16.4	-24.4	-7.6
	**Cfa**	Pampa	6.60E-03	-11.8	-19.4	-3.4
	**Cfb**	Atlantic Forest	1.06E-02	-9.4	-16.1	-2.3
		Cerrado	4.87E-02	-16.6	-30.5	-0.1
	**Cwa**	Atlantic Forest	3.03E-02	-14.7	-26.0	-1.5
	**Cwb**	-	-	-	-	-
		Amazon	2.28E-02	-7.5	-13.6	-1.1
	**Am**	Cerrado	9.20E-01	0.4	-7.6	9.1
		Amazon	2.86E-01	-3.0	-8.3	2.6
		Cerrado	4.06E-01	1.5	-2.0	5.1
	**Af**	Atlantic Forest	3.50E-01	-3.6	-10.8	4.1
		Caatinga	4.89E-01	-7.5	-26.0	15.5
	**As**	Atlantic Forest	1.02E-01	-22.8	-43.4	5.2
		Amazon	1.14E-02	-10.4	-17.7	-2.5
**0 - 30 cm**		Cerrado	1.12E-08	-10.4	-13.7	-6.9
		Atlantic Forest	3.24E-03	-19.4	-30.1	-6.9
	**Aw**	Pantanal	3.34E-51	-21.7	-24.2	-19.2
	**Bsh**	Caatinga	2.39E-01	-11.5	-27.7	8.4
		Atlantic Forest	1.79E-06	-10.7	-14.7	-6.4
	**Cfa**	Pampa	9.28E-01	0.9	-16.9	22.4
	**Cfb**	Atlantic Forest	1.85E-02	-4.4	-7.9	-0.7
		Cerrado	7.56E-01	-3.8	-24.8	23.0
	**Cwa**	Atlantic Forest	5.44E-02	-16.1	-29.8	0.3
	**Cwb**	Atlantic Forest	4.70E-38	-27.5	-30.9	-23.9

Köppen climate classification – **Tropical - Af:** Tropical rainforest; **Am:** Tropical monsoon; **As:** Tropical Savannah (winter rain), **Aw:** Tropical Savannah (summer rain); **Arid - BSh:** Arid steppe, hot; **Subtropical - Cfa:** Temperate, no dry season, hot summer; **Cfb:** Temperate, no dry season, warm summer; **Cwa:** Temperate, dry winter, hot summer; **Cwb:** Temperate, dry winter, warm summer.

Table S25 Relative change (%) in soil organic carbon stocks (SOCstocks) by soil class (Oxisol, Inceptisol, Ultisol and Entisol), stratified by biome.

Soil Class	Biome	Pval	Relative change in SOC stocks (%)	CI.lb	CI.ub
**Oxisol**	Amazon	4.4E-07	-8.53	-11.64	-5.31
	Caatinga	1.8E-01	-9.35	-21.34	4.47
	Cerrado	2.2E-07	-11.31	-15.24	-7.19
	Atlantic Forest	4.4E-08	-15.10	-19.93	-9.97
	Pampa	8.2E-02	-7.30	-14.88	0.96
**Inceptisol**	Amazon	1.5E-02	-11.98	-20.54	-2.49
	Caatinga	7.5E-02	9.08	-0.87	20.01
	Cerrado	3.3E-06	-27.61	-36.82	-17.05
	Atlantic Forest	3.9E-03	-33.05	-49.00	-12.10
**Ultisol**	Caatinga	1.8E-02	-11.27	-19.63	-2.05
	Cerrado	1.2E-01	-15.96	-32.53	4.68
	Atlantic Forest	4.4E-04	-24.34	-35.25	-11.60
	Pampa	8.8E-04	-13.41	-20.46	-5.74
	Amazon	7.6E-01	7.32	-32.20	69.89
**Entisol**	Caatinga	3.9E-02	-23.31	-40.37	-1.35
	Cerrado	3.6E-01	-9.33	-26.57	11.96
	Pampa	4.1E-01	4.96	-6.38	17.69
	Pantanal	1.0E-17	-24.98	-29.75	-19.88
	Amazon	1.2E-14	-20.38	-21.76	-18.98

Table S26 Relative change (%) in soil organic carbon stocks (SOCstocks) by agricultural system age (years), stratified by biome.

System age (years)	Biome	Pval	Relative change in SOC stocks (%)	CI.lb	CI.ub
**0 -15**	Amazon	2.3E-03	-11.3	-17.9	-4.2
**16 - 30**	Amazon	6.5E-02	-6.9	-13.7	0.5
**>30**	Amazon	2.7E-03	-8.8	-14.1	-3.1
**0 -15**	Atlantic Forest	6.5E-03	-15.0	-24.4	-4.4
**16 - 30**	Atlantic Forest	2.1E-03	-14.6	-22.8	-5.5
**>30**	Atlantic Forest	2.2E-06	-18.5	-25.1	-11.3
**0 -15**	Caatinga	1.3E-03	-17.3	-26.4	-7.1
**16 - 30**	Caatinga	8.4E-02	-14.8	-29.0	2.2
**>30**	Caatinga	7.4E-01	-6.3	-36.1	37.5
**0 -15**	Cerrado	3.1E-02	-9.4	-17.2	-0.9
**16 - 30**	Cerrado	1.6E-06	-12.4	-17.0	-7.5
**>30**	Cerrado	4.2E-03	-12.2	-19.7	-4.0
**0 -15**	Pampa	8.4E-01	1.9	-14.6	21.5
**16 - 30**	Pampa	4.9E-02	-14.5	-26.9	0.0
**>30**	Pampa	8.4E-04	-15.9	-24.0	-6.9
**0 -15**	Pantanal	1.6E-03	-24.9	-37.1	-10.3
**16 - 30**	Pantanal	3.3E-25	-23.6	-27.4	-19.6

Table S27 Summary of the meta-analysis results comparing different agricultural and management systems across all biomes.

Biome	Biome | Agri./management system	n	Relative change in SOC stocks (%)	CI.lb	CI.ub	Egger's test (p-value)
**Overall**	Native Vegetation vs. Monocropping	220	-22.2	-27.0	-17.0	8.9E-01
	Native Vegetation vs. Perennial	81	-9.9	-18.1	-0.8	1.4E-01
	Native Vegetation vs. Grassland	269	-9.4	-14.3	-4.1	5.4E-03
	Native Vegetation vs. crop rotation/ intercropping	528	-13.0	-16.8	-9.0	1.0E-04
	Native Vegetation vs. Integrated Agricultural System	172	-8.6	-13.7	-3.3	4.8E-01
	Monocropping vs. crop rotation/ intercropping	71	8.9	4.4	13.5	1.0E-04
	Monocropping vs. Integrated Agricultural System	75	12.5	5.1	20.5	1.1E-01
**Amazon**	Native Vegetation *vs.* Monocropping	17	-17.4	-27.2	-6.3	4.92E-02
	Native Vegetation *vs.* Perennial	5	-11.0	-13.8	-8.1	8.93E-01
	Native Vegetation *vs.* Grassland	59	3.9	-12.1	22.8	2.30E-03
	Native Vegetation *vs.* crop rotation/ intercropping	38	-9.3	-13.6	-4.8	2.60E-01
	Native Vegetation *vs.* Integrated Agricultural System	20	-14.0	-19.8	-7.6	6.82E-01
	Monocropping vs. crop rotation/ intercropping	8	14.1	6.5	22.4	4.62E-01
	Monocropping vs. Integrated Agricultural System	19	3.5	-2.0	9.2	9.30E-01
**Caatinga**	Native Vegetation vs. Monocropping	46	-22.7	-36.7	-5.6	1.03E-01
	Native Vegetation vs. Perennial	17	-9.3	-26.7	12.2	1.23E-01
	Native Vegetation vs. Grassland	20	-12.4	-27.5	5.7	6.33E-01
	Native Vegetation vs. crop rotation/ intercropping	18	-12.4	-27.5	5.7	5.13E-01
	Native Vegetation vs. Integrated Agricultural System	17	-8.6	-24.1	10.1	4.92E-01
	Monocropping vs. crop rotation/ intercropping	-	-	-	-	-
	Monocropping vs. Integrated Agricultural System	-	-	-	-	-
**Cerrado**	Native Vegetation vs. Monocropping	118	-15.8	-22.1	-8.9	4.09E-01
	Native Vegetation vs. Perennial	40	-10.4	-26.6	9.4	6.26E-01
	Native Vegetation vs. Grassland	122	-10.0	-16.5	-3.0	1.09E-02
	Native Vegetation vs. crop rotation/ intercropping	217	-8.1	-14.0	-1.9	8.69E-01
	Native Vegetation vs. Integrated Agricultural System	90	-7.8	-14.9	-0.1	1.00E-04
	Monocropping vs. crop rotation/ intercropping	43	8.4	2.7	14.4	1.00E-04
	Monocropping vs. Integrated Agricultural System	40	15.3	4.6	27.1	7.17E-01
**Atlantic Forest**	Native Vegetation vs. Monocropping	28	-33.0	-40.8	-24.3	5.71E-01
	Native Vegetation vs. Perennial	24	-8.8	-16.4	-0.4	8.62E-01
	Native Vegetation vs. Grassland	49	-11.4	-19.9	-2.1	2.36E-01

	Native Vegetation vs. crop rotation/ intercropping	165	-23.1	-30.0	-15.6	3.80E-03
	Native Vegetation vs. Integrated Agricultural System	27	-1.2	-7.3	5.2	7.13E-01
	Monocropping vs. crop rotation/ intercropping	12	8.6	-1.6	19.8	3.90E-01
	Monocropping vs. Integrated Agricultural System	16	3.3	-5.6	12.9	7.00E-01
**Pampa**	Native Vegetation vs. Monocropping	11	-32.1	-44.3	-17.3	1.50E-02
	Native Vegetation vs. Perennial	8	9.7	2.3	17.7	4.54E-01
	Native Vegetation vs. Grassland	-	-	-	-	-
	Native Vegetation vs. crop rotation/ intercropping	93	-15.9	-22.7	-8.6	7.00E-04
	Native Vegetation vs. Integrated Agricultural System	16	-10.0	-15.8	-3.7	4.93E-01
	Monocropping vs. crop rotation/ intercropping	8	22.0	11.8	33.1	5.94E-01
	Monocropping vs. Integrated Agricultural System	-	-	-	-	-
**Pantanal**	Native Vegetation vs. Monocropping	-	-	-	-	-
	Native Vegetation vs. Perennial	-	-	-	-	-
	Native Vegetation vs. Grassland	19	-16.3	-33.8	5.8	1.11E-02
	Native Vegetation vs. crop rotation/ intercropping	-	-	-	-	-
	Native Vegetation vs. Integrated Agricultural System	-	-	-	-	-
	Monocropping vs. crop rotation/ intercropping	-	-	-	-	-
	Monocropping vs. Integrated Agricultural System	-	-	-	-	-

Table S28 Median coefficients of variation were applied when standard deviations were unavailable, and the first (Q1) and third quartiles (Q3) were used for sensitivity analyses.

Land use (CV%)										
**Biomes**	**Soil layer (cm)**	**Crop**			**Grassland**			**Native vegetation**		
	**-**	**Q1**	**Median**	**Q3**	**Q1**	**Median**	**Q3**	**Q1**	**Median**	**Q3**
**Amazon**	0 - 10	5.8	9.1	20.6	4.3	7.2	8.2	5.2	6.5	12.4
	0 - 20	3.3	4.2	5.6	2.2	3.2	4.2	5.6	6.6	11.7
	0 - 30	3.4	4.9	7.6	5.1	6.4	9.6	3.2	4.8	10.5
**Caatinga**	0 - 10	6.0	9.5	11.6	10.5	17.3	24.7	5.0	13.3	16.4
	0 - 20	4.1	5.9	8.5	5.8	7.0	9.6	5.0	6.3	9.4
	0 - 30	3.8	4.1	5.2	3.4	3.9	4.3	4.2	5.0	5.9
**Cerrado**	0 - 10	3.0	5.1	9.6	2.4	5.2	11.9	6.0	8.9	15.5
	0 - 20	2.5	4.8	7.1	1.6	3.7	5.1	3.3	7.0	8.1
	0 - 30	2.0	2.9	4.7	3.1	4.4	5.5	3.4	4.5	7.8
**Atlantic Forest**	0 - 10	4.2	6.1	10.5	5.1	5.8	12.5	3.5	4.5	5.4
	0 - 20	2.7	3.6	6.2	6.4	7.8	9.3	2.2	2.8	4.0
	0 - 30	1.9	2.6	5.1	5.2	5.2	14.6	1.8	3.2	5.1
**Pampa**	0 - 10	2.4	6.0	6.5	*	*	*	***	***	***
	0 - 20	0.7	2.8	6.4	*	*	*	4.1	6.3	8.5
	0 - 30	1.0	1.3	1.6	*	*	*	4.5	4.8	5.0
**Pantanal**	0 - 10	-	-	-	**	**	**	**	**	**
	0 - 20	-	-	-	**	**	**	**	**	**
	0 - 30	-	-	-	**	**	**	**	**	**

* Median CV from agriculture used when mean and SD were not reported in grassland
** Median Cerrado CVs applied for lack of Pantanal SD
*** In the absence of references for a specific soil layer, we adopted the SD values reported for the 0–20 cm layer.

S2. Supplementary Figures

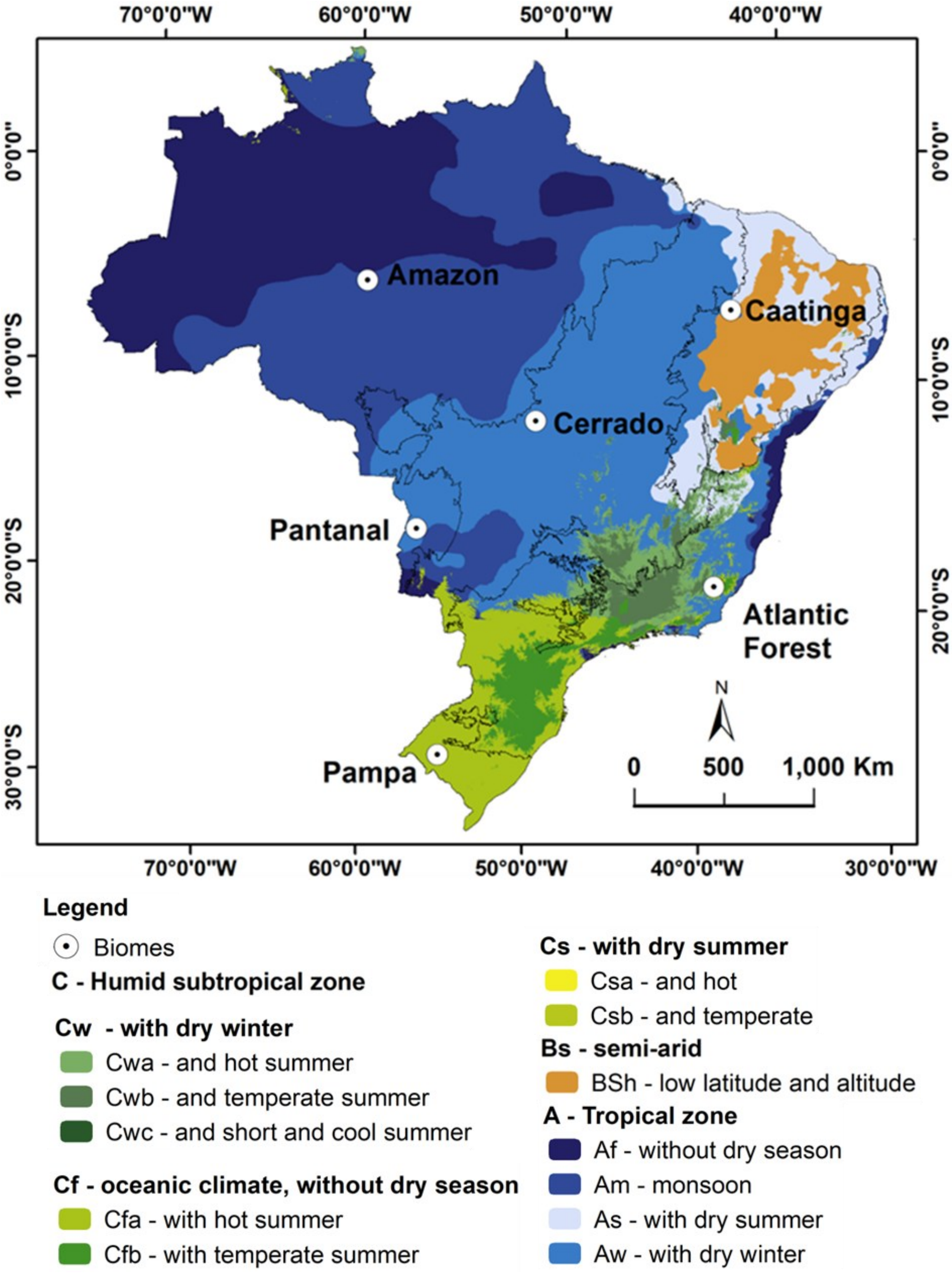

Fig. S1 Climate classification across Brazilian biomes based on Köppen criteria. The map shows the main Brazilian biomes (Amazon, Cerrado, Caatinga, Atlantic Forest, Pantanal and Pampa) overlaid with Köppen climate zones and subtypes. Tropical areas (blue shades) include Af (no dry season), Am (monsoon), As (dry summer) and Aw (dry winter). Semi-arid regions (orange) correspond to BSh. The humid subtropical zone (green shades) includes Cfa and Cfb (oceanic climate without a dry season) and Cwa, Cwb and Cwc (dry winter).Areas with dry summer are shown in yellow (Csa and Csb).The free biome boundary data were obtai ned from IBGE (https://www.ibge.gov.br/geociencias/todos-os-produtos geociencias.html), and the climate-classification raster used as the base layer was derived from zoning proposed by Alvares et al. 2013[1] (https://www.schweizerbart.de/papers/metz/detail/22/82078/Koppen_s_climate_classification_map_for_Brazil), also available at www.ipef.br/geodatabase/.

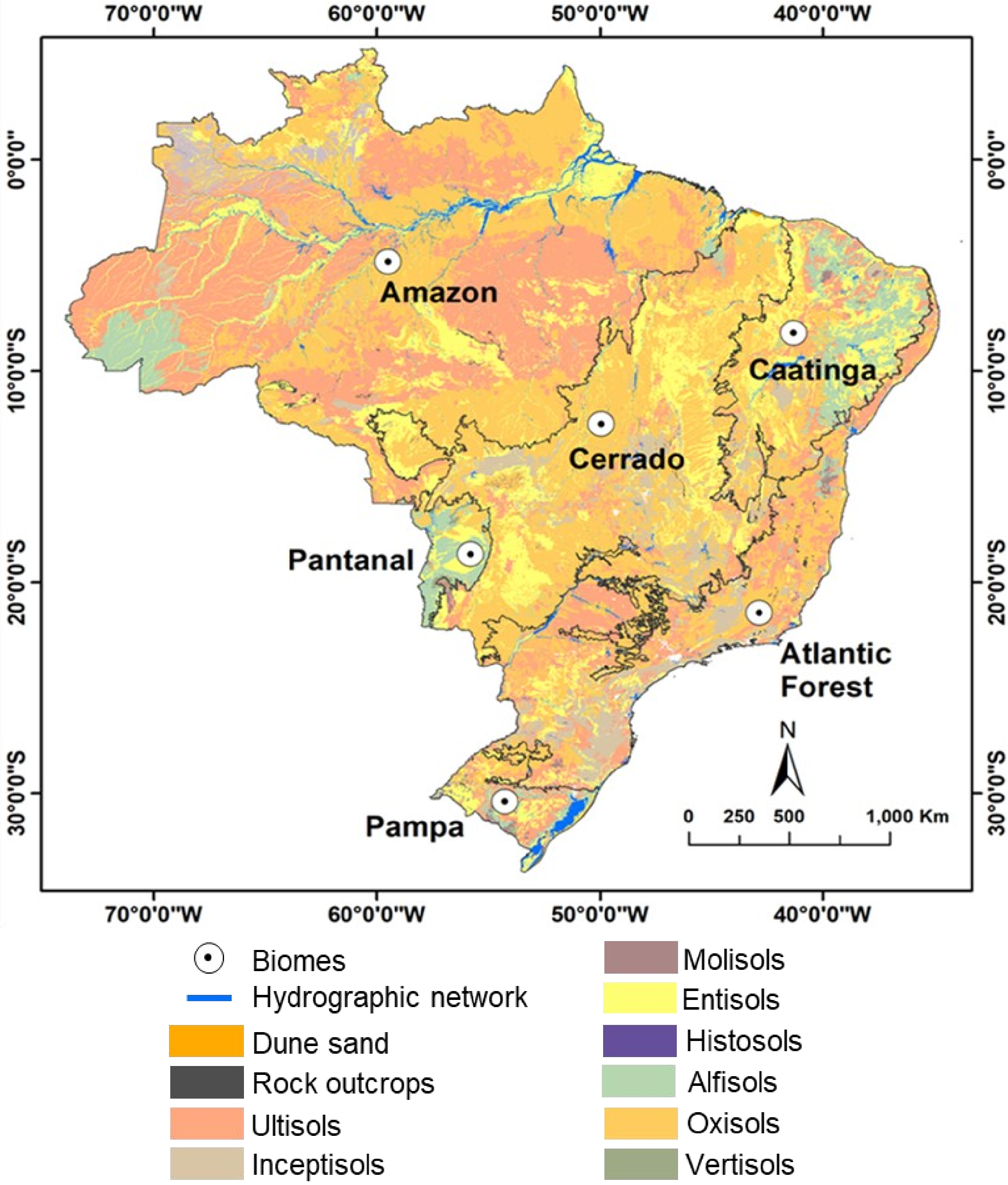

Fig. S2 Brazilian soil classes across biomes according to the U.S. Soil Taxonomy. The map shows the distribution of the main Brazilian biomes (Amazon, Cerrado, Caatinga, Atlantic Forest, Pantanal and Pampa) and their relationship with soil classes: water bodies (blue), dune sands (light yellow), rock outcrops (gray), Entisols (yellow), Inceptisols (orange), Alfisols (green), Ultisols (salmon), Oxisols (ochre), Mollisols (brown) and Vertisols (olivegreen). The free biome boundary data were obtained from IBGE (https://www.ibge.gov.br/geociencias/todos-os-produtos-geociencias.html), and the Brazilian soil class shapefile was obtained from the adapted Soil Map of Brazil Embrapa[2], available at https://geoinfo.dados.embrapa.br/catalogue/#/dataset/1688.

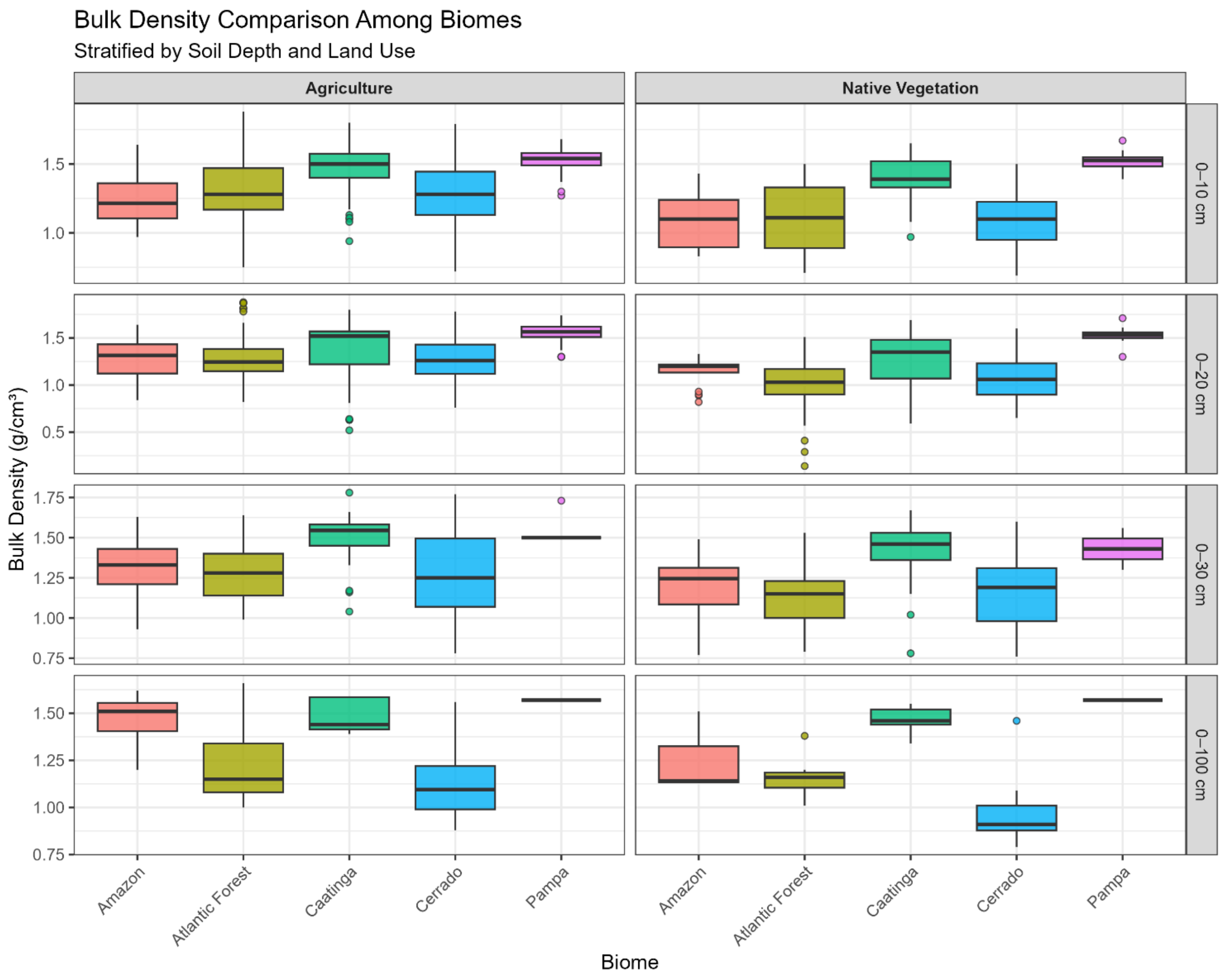

Fig. S3 Bulk density across Brazilian biomes under agriculture and native vegetation. Boxplots show bulk density (g cm^{-3}) across five biomes (Amazon, Atlantic Forest, Caatinga, Cerrado, and Pampa) for four soil layers (0–10, 0–20, 0–30, and 0–100 cm), separately for agriculture (left panels) and native vegetation (right panels). For each boxplot, the central line represents the median, the box spans the interquartile range (IQR), and the whiskers extend to 1.5 × IQR. Points outside this range represent outliers.

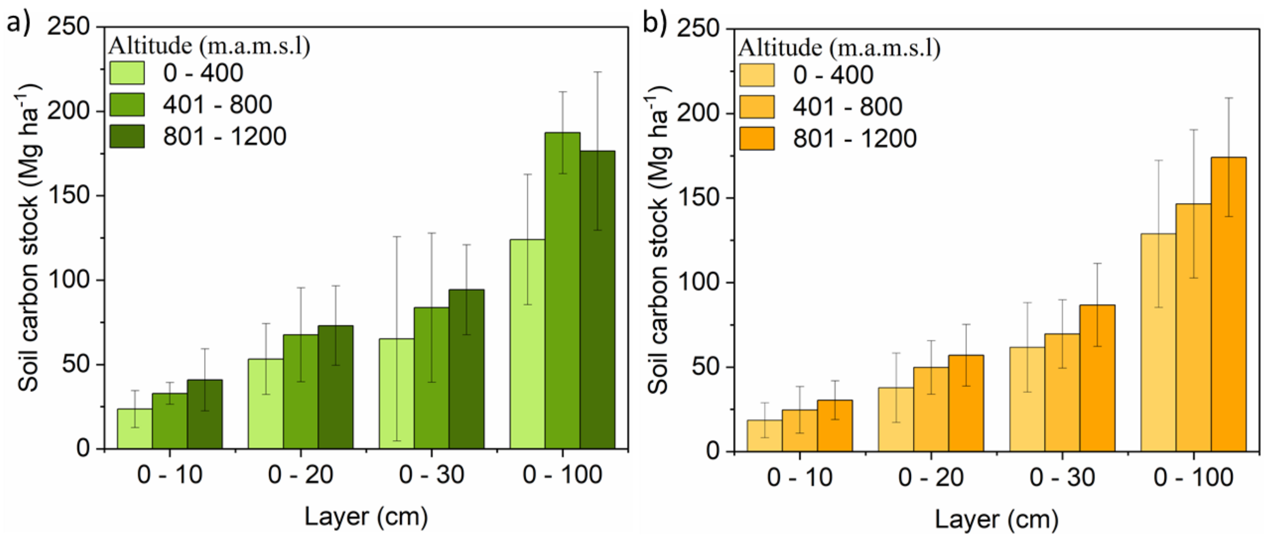

Fig. S4 SOC$_{stocks}$ under native vegetation and agriculture across altitudes in the Atlantic Forest. Panels a and b show the mean soil organic carbon stocks (SOC$_{stocks}$: Mg ha^{-1}) across four soil layers (0–10, 0–20, 0–30, and 0–100 cm) along an altitudinal gradient in the Atlantic Forest for native vegetation (a), represented by green bars, and agriculture (b), represented by orange bars. Bars represent mean SOC$_{stocks}$, and error bars indicate the standard deviations.

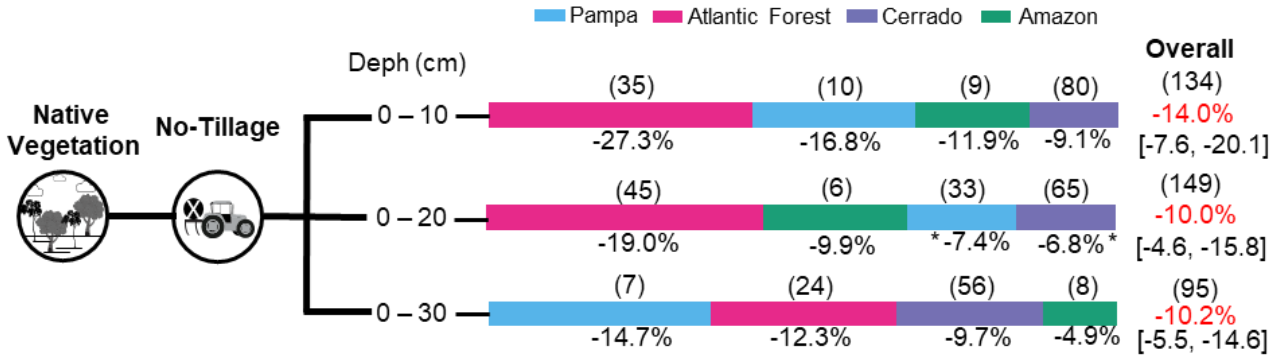

Fig. S5 Assessment of the stratification effect in the no-tillage system across soil layers. The bars represent the mean effect sizes expressed as percent change (±95% CI of the response ratio, lnRR), estimated using a random-effects model. Values in parentheses indicate the number of paired observations used to calculate changes in SOC$_{stocks}$ between native vegetation and no-tillage systems, whereas values in brackets indicate the confidence intervals. The notation ns indicates non-significant statistical differences at the 95% confidence level. In the overall panel, negative effects are shown in red.

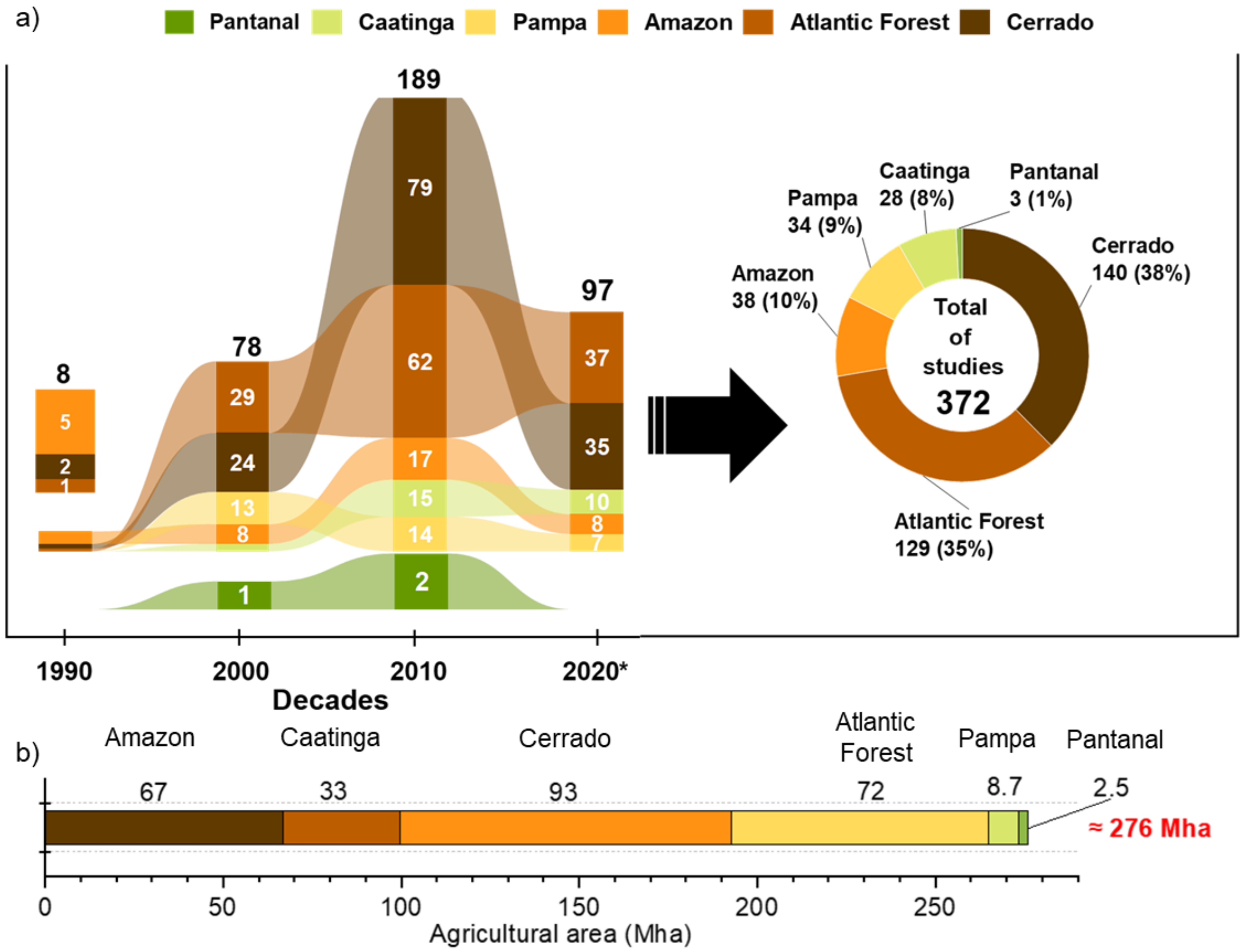

Fig. S6 Three-decade trends in SOC_{stock} studies and corresponding agricultural land area across Brazilian biomes. a) Temporal evolution of studies reporting soil organic carbon (SOC) stocks in Brazil over the past three decades, disaggregated by biome (Amazon, Cerrado, Atlantic Forest, Caatinga, Pampa and Pantanal). The stacked density ribbons illustrate both the magnitude and distribution of published studies, with numeric labels indicating the count of studies per biome and decade. The accompanying donut chart summarizes the proportional contribution of each biome to the total number of SOC_{stock} studies. b) Agricultural land area (Mha) associated with each Brazilian biome, highlighting their relative contributions to the national agricultural footprint. Values above the bar indicate the estimated agricultural extent for each biome, summing to approximately 276 Mha.[3].

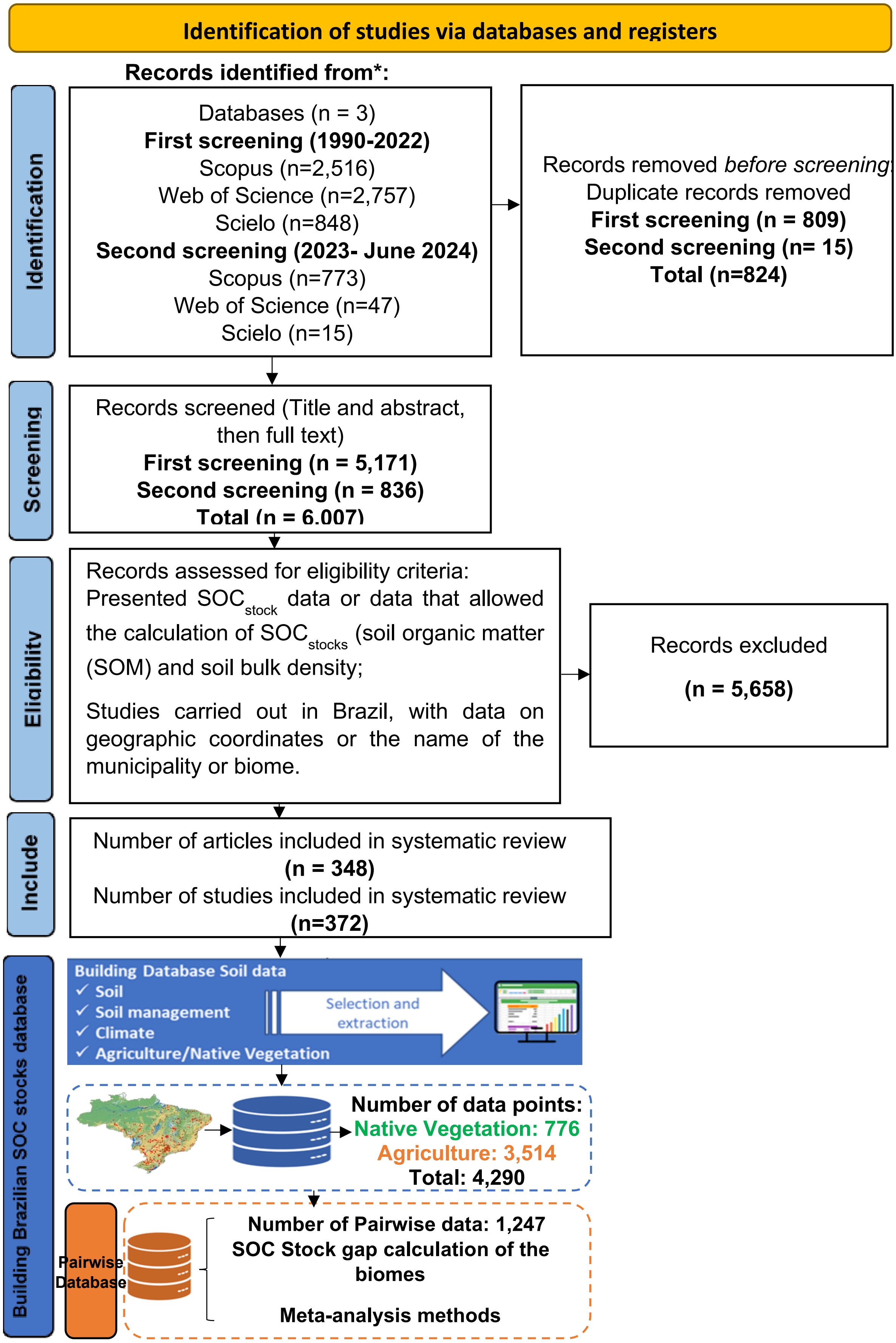

Fig. S7 PRISMA flow diagram of the systematic review and database construction. The template used to prepare the PRISMA flow diagram was obtained from Page et al.7 (https://guides.lib.unc.edu/ld.php?content_id=61167715).

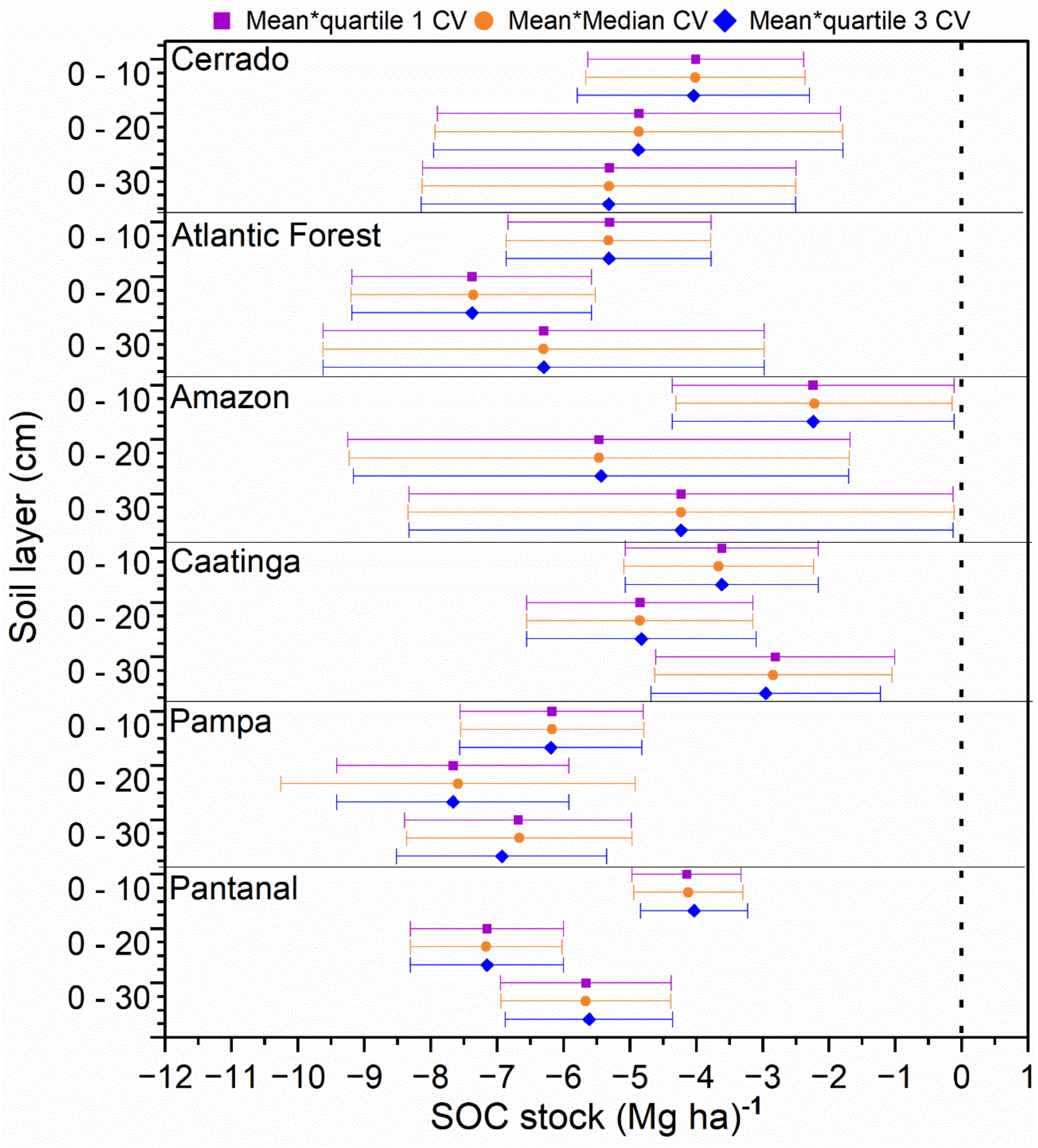

Fig. S8 Sensitivity analysis of SOC_{stock} gaps under alternative SD-imputation strategies. The figure presents a sensitivity analysis assessing the robustness of the SOC stock gaps meta-analysis to the imputation of missing standard deviations (SDs). Three SD-imputation strategies are compared, each derived from the mean coefficient of variation (CV) at different distributional points: the first quartile (purple), the median (orange), and the third quartile (blue).

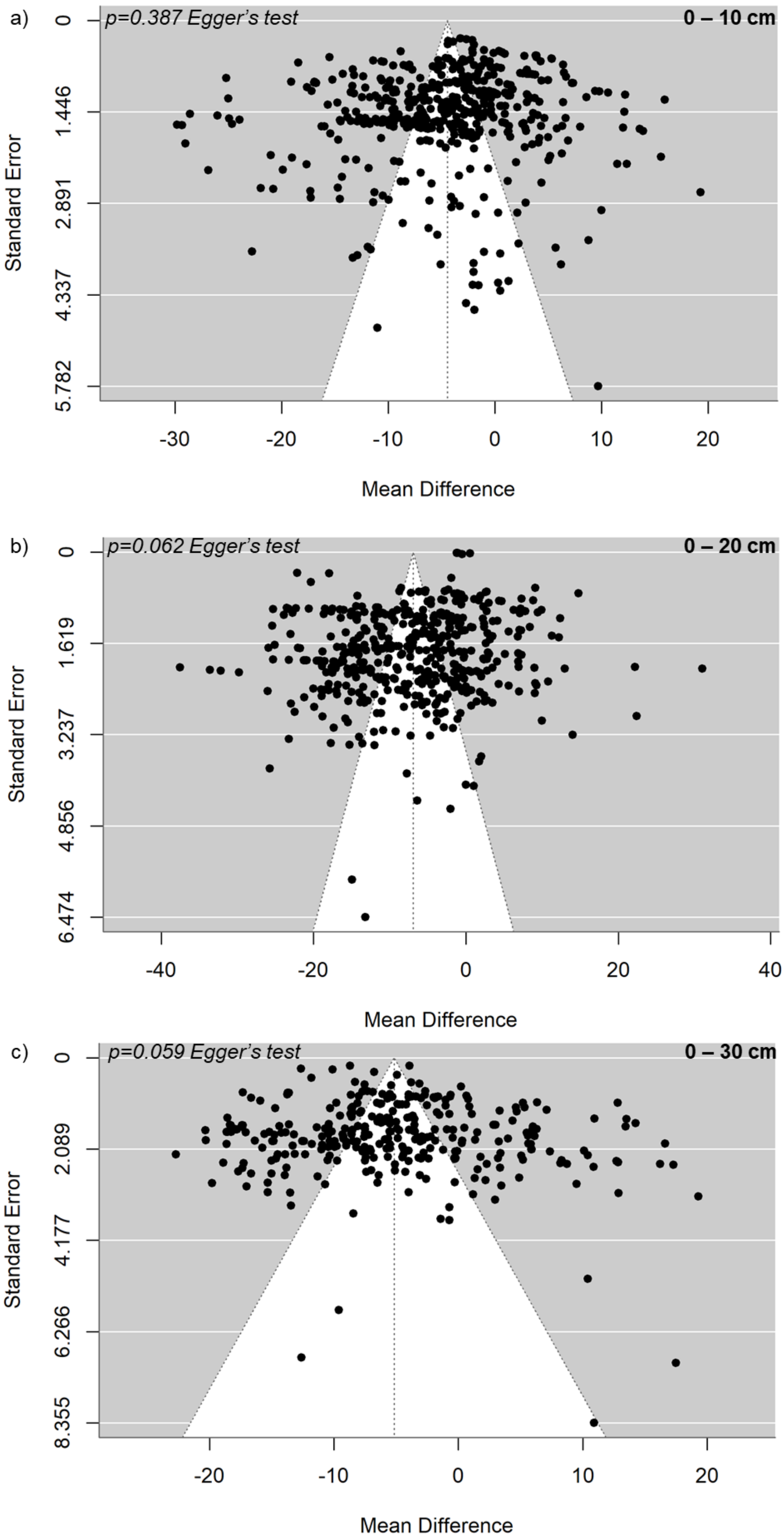

Fig. S9 Assessments of publication bias under effect land use change (Native Vegetation to Agriculture) on SOC_{stock}. Funnel plot of effect sizes by Egger's publication bias method for soil carbon stock (SOC_{stock}) under land use change considering different soil layers overall: (a) 0–10 cm, (b) 0–20 cm, and (c) 0–30 cm.The absence of publication bias is indicated when the P value > 0.05. In this condition, at least 95% of the studies must be within the triangular region and evenly distributed on both sides of the vertical line.

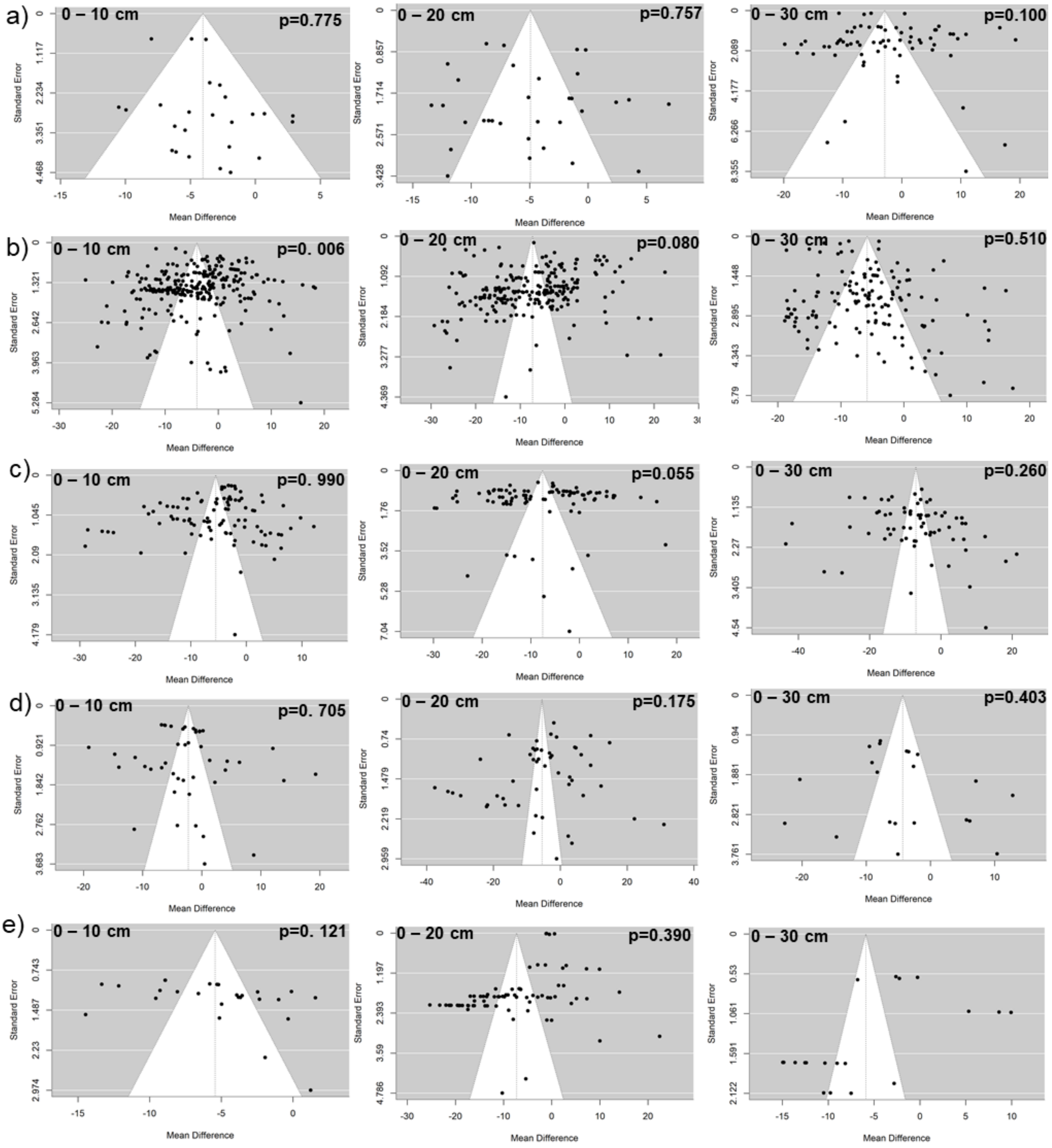

Fig. S10 Publication bias in land-use change from native vegetation to agriculture on soil organic carbon stocks. Analyses were conducted for the Amazon (a), Caatinga (b), Cerrado (c), Atlantic Forest (d), and Pampa (e) biomes across soil layers of 0–10, 0–20, and 0–30 cm. The absence of publication bias is indicated when P > 0.05; under this condition, at least 95% of the studies fall within the triangular region and are evenly distributed on both sides of the vertical line. The Pantanal biome did not present sufficient data (<10 observations) to perform the Egger test. An asterisk (*) indicates a non-significant difference.

S3. Supplementary Discussion

The Cerrado and Atlantic Forest biomes presented the highest numbers of studies, with 140 and 129 units, respectively, approximately 73% of the total of 372 studies (Fig.S4a). The Amazon recorded 38 studies, followed by the Pampa with 34. The Caatinga and Pantanal biomes presented the lowest values, with 28 and 3 studies, respectively. Regarding the density of studies per available agricultural area (276 Mha) Mha, Brazil has 1 study for every 0.7 Mha (Fig.S4b). There has been a significant increase in the number of studies over the last three decades, with a growth of up to 9 times between the 1990s (8) and 2000s (78) and between 2000 and 2010 (189), a significant increase of 143% in the number of studies (Fig.S4a). A noteworthy number of studies have been carried out up to the first half of the 2020s (97), which could surpass the number of studies from the previous decade if the same trend continues for the second half.

Many studies (except Pantanal) have been conducted, mainly in the Cerrado and Atlantic Forest, which are home to ≈60% of Brazil's agricultural area. Our results revealed the great potential of the available information on SOC stocks in the country. This represents an important step towards the establishment of its regulated C market, considered strategic in its climate mitigation policy.

Some events, such as the creation of the United Nations Framework Convention on Climate Change (UNFCCC), post-Eco 92; the international recognition of soil's potential for climate mitigation; and the launch of important initiatives ("4p1000" and "RECSOIL")[4,5,6], may explain the successive increases in the number of studies observed.

S4. Supplementary references

1. Alvares, C. A. et al. Köppen's climate classification map for Brazil. Meteorol. Z. **22**, 711–728(2013).
2. Santos, H.G et al. O Novo Mapa de Solos do Brasil Legenda Atualizada. Rio de Janeiro, Embrapa, (2011).
3. Projeto MapBiomas – Coleção [9] da Série Anual de Mapas de Cobertura e Uso da Terra do Brasil, accessed on [8/2024] via the link: https://plataforma.brasil.mapbiomas.org/cobertura
4. Rumpel, C., Lehmann, J., & Chabbi, A..'4 per 1,000'initiative will boost soil carbon for climate and food security. *Nat.* **553**, 7686 (2018).
5. Food and Agriculture organisation of the United Nations (FAO): Recarbonization of Global Soils - A dynamic response to offset global emissions, FAO, (2019).
6. Amelung, W. et al. Towards a global-scale soil climate mitigation strategy. *Nat. Commun.* **11**, 5427 (2020).
7. Page, M. J. et al. The PRISMA 2020 statement: an updated guideline for reporting systematic reviews. *BMJ* n71 (2021).

S5. Supplementary Database References

1. Abrell, T. *et al*. Shifting cultivation in decline: An analysis of soil fertility and weed pressure in intensified cropping systems in Eastern Amazon. *Agric. Ecosyst*. Environ **360**, 108793 (2024).

2. Ajayi, A. E. *et al*. Long-term no tillage management impact on soil hydro-physical properties in coffee cultivation. *Geoderma* **404**, 115306 (2021).

3. Alburquerque, M. A. *et al*. Carbon and nitrogen in a F erralsol under zero-tillage rotations based on cover, cash or hay crops. *Soil Use Manag* **31**, 1–9 (2015).

4. Almeida, E. M., Campelo Júnior, J. H. & Finger, Z. Determinação do estoque de carbono em Teca (Tectona grandis L. F.) em diferentes idades. *Ciênc. Florest* **20**, 559–568 (2010).

5. Almeida, L. L. D. S. *et al*. Soil carbon and nitrogen stocks and the quality of soil organic matter under silvopastoral systems in the Brazilian Cerrado. *Soil Tillage Res* **205**, 104785 (2021).

6. Almeida, V. P. D., Alves, M. C., Silva, E. C. D. & Oliveira, S. A. D. Rotação de culturas e propriedades físicas e químicas em Latossolo Vermelho de Cerrado sob preparo convencional e semeadura direta em adoção. *Rev. Bras. Ciênc. Solo* **32**, 1227–1237 (2008).

7. Alves, L. A. *et al*. The effect of crop rotation and sheep grazing management on plant production and soil C and N stocks in a long-term integrated crop-livestock system in Southern Brazil. *Soil Tillage Res* **203**, 104678 (2020).

8. Alves, R. P., Couto Junior, A. F., Martins, E. D. S. & Nardoto, G. B. Role of soil carbon in the landscape functioning of the Alto São Bartolomeu watershed in the Cerrado region, Brazil. *Pesqui. Agropecuária Bras* **51**, 1241–1251 (2016).

9. Andrade, A. P. *et al*. Physical properties of a humic cambisol under tillage and cropping systems after 12 years. *Rev. Bras. Ciênc. Solo* **34**, 219-226 (2010).

10. Anésio, A. H. C. *et al*. Management of marandu grass pasture increases soil carbon and nitrogen stocks in forest grazing systems in the Brazilian Cerrado. *Rev Bras Cienc Solo **48**, e0240057* (2024).

11. Rodrigues, L. A. T. *et al*. Carbon saturation deficit and litter quality drive the stabilization of litter-derived C in mineral-associated organic matter in long-term no-till soil. *CATENA* **219**, 106590 (2022).

12. Araújo Filho, R. N. D. *et al*. Recovery of carbon stocks in deforested caatinga dry forest soils requires at least 60 years. *For. Ecol. Manag* **407**, 210–220 (2018).

13. Araújo, F. S. *et al*. Fertility and total organic carbon in oxisol under different management systems in savannah of Piauí, Brazil. *Trop. Subtrop. Agroecosystems* **20**, 165 -172 (2017).

14. Araújo, J.K.S. *et al*. Umbric Ferralsols along a climosequence from the Atlantic coast to the highlands of northeastern Brazil: Characterization and carbon mineralization. *Geoderma* **293**, 34-43 (2017).

15. Araújo, L. G., Figueiredo, C. C. & Sousa, D. M. G. Gypsum application increases the carbon stock in soil under sugar cane in the Cerrado region of Brazil. *Soil Res* **55**, 38-46 (2017).

16. Assis, C. P. D., Jucksch, I., Mendonça, E. D. S. & Neves, J. C. L. Carbono e nitrogênio em agregados de Latossolo submetido a diferentes sistemas de uso e manejo. *Pesqui. Agropecuária Bras* **41**, 1541–1550 (2006).

17. Assmann, J. M. et al. Soil carbon and nitrogen stocks and fractions in a long-term integrated crop–livestock system under no-tillage in southern Brazil. *Agric. Ecosyst. Environ* **190**, 52–59 (2014).

18. Assunção, S. A., Pereira, M. G., Rosset, J. S., Berbara, R. L. L. & García, A. C. Carbon input and the structural quality of soil organic matter as a function of agricultural management in a tropical climate region of Brazil. *Sci. Total Environ* **658**, 901–911 (2019).

19. Babujia, L. C., Hungria, M., Franchini, J. C. & Brookes, P. C. Microbial biomass and activity at various soil depths in a Brazilian oxisol after two decades of no-tillage and conventional tillage. *Soil Biol. Biochem* **42**, 2174–2181 (2010).

20. Balieiro, F. D. C. *et al.* Fertilidade e carbono do solo e uso da água pelo eucalipto numa topossequencia em Seropédica, RJ. *Rev. Árvore* **32**, 153–162 (2008).

21. Barros, J. D. S. *et al.* Carbon and nitrogen stocks under different management systems in the Paraiban "Sertão". Afriacan. *J.Res. Agric* **10**, 130-136 (2015).

22. Barros, J. D. S. *et al.* Estoque de carbono e nitrogênio em sistemas de manejo do solo, nos tabuleiros costeiros Paraibanos. *Rev. Caatinga* **23**, 35-42 (2013).

23. Batista, I. *et al.* Teores e estoque de carbono em frações lábeis e recalcitrantes da matéria orgânica do solo sob integração lavoura-pecuária no bioma Cerrado. *Semina: Ciênc. Agra* **34**, 3377-3388 (2013).

24. Batjes, N. H. & Batjes, N. H. Organic carbon stocks in the soils of Brazil. *Soil Use Manag* **21**, 22-24 (2005).

25. Bayer, C., Martin-Neto, L., Mielniczuk, J. & Pavinato, A. Carbon storage in labile fractions of soil organic matter in a tropical no-tillage Oxisol. *Pesqui. Agropecuária Bras* **39**, 677–683 (2004).

26. Bayer, C., Martin-Neto, L., Mielniczuk, J., Dieckow, J. & Amado, T. J. C. C and N stocks and the role of molecular recalcitrance and organomineral interaction in stabilizing soil organic matter in a subtropical Acrisol managed under no-tillage. *Geoderma* **133**, 258–268 (2006).

27. Bayer, C., Martin-Neto, L., Mielniczuk, J., Pavinato, A. & Dieckow, J. Carbon sequestration in two Brazilian Cerrado soils under no-till. *Soil Tillage Res* **86**, 237–245 (2006).

28. Bayer, C., Mielniczuk, J., Amado, T. J. C., Martin-Neto, L. & Fernandes, S. V. Organic matter storage in a sandy clay loam Acrisol affected by tillage and cropping systems in southern Brazil. *Soil Tillage Res* **54**, 101–109 (2000).

29. Bernardi, A. C. D. C. *et al.* Carbon and nitrogen stocks of an Arenosol under irrigated fruit orchards in semiarid Brazil. *Sci. Agric* **64**, 169–175 (2007).

30. Bieluczyk, W. *et al.* Linking above and belowground carbon sequestration, soil organic matter properties, and soil health in Brazilian Atlantic Forest restoration. *J. Environ. Manage* **344**, 118573 (2023).

31. Bieluczyk, W. *et al.* Soil carbon and nitrogen stocks, light organic matter, and remaining phosphorus under a crop-livestock integration system. *Semina Ciênc. Agrár* **38**, 1825 (2017).

32. Bispo, D. F. A. *et al.* Soil Organic Carbon as Response to Reforestation Age and Land Use Changes: A Qualitative Approach to Ecosystem Services. *Sustainability* **15**, 6863 (2023).

33. Blanchart, E. *et al.* Effect of Direct Seeding Mulch-Based Systems on Soil Carbon Storage and Macrofauna in Central Brazil. *Agric. conspec. sci* **72**, 81-87 (2007).

34. Boddey, R. M. *et al.* Carbon accumulation at depth in Ferralsols under zero-till subtropical agriculture. *Glob. Change Biol* **16**, 784–795 (2010).

35. Bona, F. D. D., Bayer, C., Bergamaschi, H. & Dieckow, J. Carbono orgânico no solo em sistemas irrigados por aspersão sob plantio direto e preparo convencional. *Rev. Bras. Ciênc. Solo* **30**, 911–919 (2006).

36. Bordonal, R. D. O. *et al.* Changes in quantity and quality of soil carbon due to the land-use conversion to sugarcane (Saccharum officinarum) plantation in southern Brazil. Agric. Ecosyst. Environ **240**, 54–65 (2017).

37. Bordonal, R. D. O. *et al*. Sugarcane yield and soil carbon response to straw removal in south-central Brazil. Geoderma **328**, 79–90 (2018).

38. Brandani, C. B., Abbruzzini, T. F., Conant, R. T. & Cerri, C. E. P. Soil organic and organomineral fractions as indicators of the effects of land management in conventional and organic sugar cane systems. *Soil Res* **55**, 145-161 (2017).

39. Braz, S. P. *et al*. Soil Carbon Stocks under Productive and Degraded Brachiaria Pastures in the Brazilian Cerrado. *Soil Sci. Soc. Am. J* **77**, 914–928 (2013).

40. Brevilieri, R. C. *et al*. No-tillage and fertilization effectively improved soil carbon and nitrogen in a subtropical Ferralsol. *Soil Tillage Res*. **241**, 106095 (2024).

41. Briedis, C. *et al*. Changes in Organic Matter Pools and Increases in Carbon Sequestration in Response to Surface Liming in an Oxisol under Long-Term No-Till. *Soil Sci. Soc. Am. J* **76**, 151–160 (2012).

42. Briedis, C. *et al*. How does no-till deliver carbon stabilization and saturation in highly weathered soils? *CATENA* **163**, 13–23 (2018).

43. Briedis, C. *et al*. Preservation of labile organic compounds is the pathway for carbon storage in a 23-year continuous no-till system on a Ferralsol in southern Brazil. *Geoderma Reg* **33**, e00643 (2023).

44. Buurman, P. & Roscoe, R. Different chemical composition of free light, occluded light and extractable SOM fractions in soils of Cerrado and tilled and untilled fields, Minas Gerais, Brazil: a pyrolysis-GC/MS study. *Eur. J. Soil Sci* **62**, 253–266 (2011).

45. Cá, J., Lustosa Filho, J. F., Da Silva, N. R., De Castro, C. R. T. & De Oliveira, T. S. C and N stocks in silvopastoral systems with high and low tree diversity: Evidence from a twenty-two year old field study. *Sci. Total Environ* **833**, 155298 (2022).

46. Calegari, A. et al. Impact of Long-Term No-Tillage and Cropping System Management on Soil Organic Carbon in an Oxisol: A Model for Sustainability. *Agron. J* **100**, 1013–1019 (2008).

47. Camacho, P. A. G. *et al*. Intensification of Pasture-Based Animal Production System Has Little Short-Term Effect on Soil Carbon Stock in the Southern Brazilian Highland. *Agronomy* **13**, 850 (2023).

48. Campos, B.-H. C. D., Amado, T. J. C., Bayer, C., Nicoloso, R. D. S. & Fiorin, J. E. Carbon stock and its compartments in a subtropical oxisol under long-term tillage and crop rotation systems. *Rev. Bras. Ciênc. Solo* **35**, 805–817 (2011).

49. Campos, L. P., Leite, L. F. C., Maciel, G. A., Brasil, E. L. & Iwata, B. D. F. Estoques e frações de carbono orgânico em Latossolo Amarelo submetido a diferentes sistemas de manejo. *Pesqui. Agropecuária Bras* **48**, 304–312 (2013).

50. Cardoso, E. L., Silva, M. L. N., Silva, C. A., Curi, N. & Freitas, D. A. F. D. Estoques de carbono e nitrogênio em solo sob florestas nativas e pastagens no bioma Pantanal. *Pesqui. Agropecuária Bras* **45**, 1028–1035 (2010).

51. Carlos, F. S. *et al*. Long-term cover crops and no-tillage in Entisol increase enzyme activity and carbon stock and enable the system fertilization in southern Brazil. *Geoderma Reg* **34**, e00700 (2023).

52. Carmeis Filho, A. C. A. *et al*. Changes in Soil Physical Properties and Carbon Protection Mechanisms by Surface Application of Lime in a Tropical No-Tillage System. *Soil Sci. Soc. Am. J* **82**, 56–65 (2018).

53. Carneiro, M. A. C. *et al*. Atributos indicadores de qualidade em solos de Cerrado no entorno do parque nacional das Emas, Goiás. *Biosci. J* **29**, 1857-1857 (2013).

54. Carvalho Leite, L. F. *et al*. Simulating trends in soil organic carbon of an Acrisol under no-tillage and disc-plow systems using the Century model. *Geoderma* **120**, 283–295 (2004).

55. Carvalho, A. M. D., Marchão, R. L., Souza, K. W. & Bustamante, M. M. D. C. Soil fertility status, carbon and nitrogen stocks under cover crops and tillage regimes. *Rev. Ciênc. Agronômica* **45**, 914–921 (2014).

56. Carvalho, E. J. M. *et al.* Comportamento físico-hídrico de um podzólico vermelho-amarelo câmbico fase terraço sob diferentes sistemas de manejo. *Pesq. agropec. bras*. **34**, 257-265 (1999).

57. Carvalho, J. L. N. *et al.* Carbon sequestration in agricultural soils in the Cerrado region of the Brazilian Amazon. *Soil Tillage Res*. **103**, 342–349 (2009).

58. Carvalho, J. L. N. *et al.* Crop-pasture rotation: A strategy to reduce soil greenhouse gas emissions in the Brazilian Cerrado. *Agric. Ecosyst. Environ* **183**, 167–175 (2014).

59. Carvalho, J. L. N. *et al.* Impact of pasture, agriculture and crop-livestock systems on soil C stocks in Brazil. *Soil Tillage Res* **110**, 175–186 (2010).

60. Cavalcante, D. M., Silva, I. R. D. & Oliveira, T. S. D. Soil quality indicators for monitoring the short-term effects of mined soil rehabilitation strategies for bauxite. R*ev. Bras. Ciênc. Solo* **47**, e0220126 (2023).

61. Ceddia, M. B., Villela, A. L. O., Pinheiro, É. F. M. & Wendroth, O. Spatial variability of soil carbon stock in the Urucu river basin, Central Amazon-Brazil. *Sci. Total Environ* **526**, 58–69 (2015).

62. Cerri, C. C. *et al.* Canne à sucre et sequestration du carbone sugarcane and carbon sequestration. *Académie d'Agriculture de France*. 1-15 (2004).

63. Cerri, C. C. *et al.* Effect of sugarcane harvesting systems on soil carbon stocks in Brazil: an examination of existing data. *Eur. J. Soil Sci* **62**, 23–28 (2011).

64. Cerri, C. E. P. *et al.* Modeling Soil Carbon from Forest and Pasture Ecosystems of Amazon, Brazil. *Soil Sci. Soc. Am. J* **67**, 1879–1887 (2003).

65. Chapuis Lardy, L., Brossard, M., Lopes Assad, M. L. & Laurent, J.-Y. Carbon and phosphorus stocks of clayey Ferralsols in Cerrado native and agroecosystems, Brazil. *Agric. Ecosyst. Environ* **92**, 147–158 (2002).

66. Cherubin, M. R., Tormena, C. A. & Karlen, D. L. Soil Quality Evaluation Using the Soil Management Assessment Framework (SMAF) in Brazilian Oxisols with Contrasting Texture. *Rev. Bras. Ciênc. Solo* **41**, (2017).

67. Conceição, M. C. G. D. *et al.* Changes in Soil Carbon Stocks under Integrated Crop-Livestock-Forest System in the Brazilian Amazon Region. *Agric. Sci* **8**, 904–913 (2017).

68. Conceição, P. C., Amado, T. J. C., Mielniczuk, J. & Spagnollo, E. Qualidade do solo em sistemas de manejo avaliada pela dinâmica da matéria orgânica e atributos relacionados. *Rev. Bras. Ciênc. Solo* **29**, 777–788 (2005).

69. Conceição, P. C., Dieckow, J. & Bayer, C. Combined role of no-tillage and cropping systems in soil carbon stocks and stabilization. *Soil Tillage Res* **129**, 40–47 (2013).

70. Cook, R. L., Binkley, D., Mendes, J. C. T. & Stape, J. L. Soil carbon stocks and forest biomass following conversion of pasture to broadleaf and conifer plantations in southeastern Brazil. *For. Ecol. Manag* **324**, 37–45 (2014).

71. Cooper, H. V. *et al.* Long-term zero-tillage enhances the protection of soil carbon in tropical agriculture. *Eur. J. Soil Sci* **72**, 2477–2492 (2021).

72. Corazza, E. J., Silva, J. E., Resck, D. V. S. & Gomes, A. C. Comportamento de diferentes sistemas de manejo como fonte ou depósito de carbono em relação à vegetação de Cerrado. *Rev. Bras. Ciênc. Solo* **23**, 425–432 (1999).

73. Corbeels, M. *et al.* Soil carbon storage potential of direct seeding mulch-based cropping systems in the Cerrados of Brazil. *Glob. Change Biol* **12**, 1773–1787 (2006).

74. Corrêa, J. C. *et al.* Carbon fractions and stock in response to solid and fluid organomineral fertilizers in highly fertile soils. *Pesqui. Agropecuária Bras* **54**, e00266 (2019).

75. Corrêa, R. S. *et al.* Fluxos de óxido nitroso e suas relações com atributos físicos e químicos do solo. *Pesqui. Agropecuária Bras* **51**, 1148–1155 (2016).

76. Coser, T. R. *et al.* Short-term buildup of carbon from a low-productivity pastureland to an agrisilviculture system in the Brazilian savannah. *Agric. Syst* **166**, 184–195 (2018).

77. Costa Junior, C. *et al.* Assessing soil carbon storage rates under no-tillage: Comparing the synchronic and diachronic approaches. *Soil Tillage Res* **134**, 207–212 (2013).

78. Costa, A. A., Dias, B. D. O., Fraga, V. D. S., Santana, C. C. & Silva, N. D. Carbon and nitrogen stocks in soils under different forms of use in the Cerrado. *Rev. Bras. Eng. Agríc. E Ambient* **24**, 528–533 (2020).

79. Costa, F. D. S., Bayer, C., Zanatta, J. A. & Mielniczuk, J. Estoque de carbono orgânico no solo e emissões de dióxido de carbono influenciadas por sistemas de manejo no sul do Brasil. *Rev. Bras. Ciênc. Solo* **32**, 323–332 (2008).

80. Costa, N. R. *et al.* Atributos do Solo e Acúmulo de Carbono na Integração Lavoura-Pecuária em Sistema Plantio Direto. *Rev. Bras. Ciênc. Solo* **39**, 852–863 (2015).

81. Costa, O. V. *et al.* Cobertura do solo e degradação de pastagens em área de domínio de Chernossolos no sul da Bahia. *Rev. Bras. Ciênc. Solo* **24**, 843–856 (2000).

82. Costa, O. V. *et al.* Estoque de carbono do solo sob pastagem em área de tabuleiro costeiro no sul da Bahia. *Rev. Bras. Ciênc. Solo* **33**, 1137–1145 (2009).

83. Cruz, A. C. R., Pauletto, E. A., Flores, C. A. & Silva, J. B. Atributos físicos e carbono orgânico de um Argissolo Vermelho sob sistemas de manejo. *Rev. Bras. Ciênc. Solo* **27**, 1105–1112 (2003).

84. Cunha, E. D. Q. *et al.* Sistemas de preparo do solo e culturas de cobertura na produção orgânica de feijão e milho: II - atributos biológicos do solo. *Rev. Bras. Ciênc. Solo* **35**, 603–611 (2011).

85. d'Andréa, A. F., Silva, M. L. N., Curi, N. & Guilherme, L. R. G. Estoque de carbono e nitrogênio e formas de nitrogênio mineral em um solo submetido a diferentes sistemas de manejo. *Pesqui. Agropecuária Bras* **39**, 179–186 (2004).

86. Da Luz, F. B. *et al.* Monitoring soil quality changes in diversified agricultural cropping systems by the Soil Management Assessment Framework (SMAF) in southern Brazil. *Agric. Ecosyst. Environ* **281**, 100–110 (2019).

87. Da Silva Farias, P. G. *et al.* Physical fractions of organic matter and mineralizable soil carbon as quality indicators in areas under different forms of use in the Cerrado-Pantanal Ecotone. *Environ. Monit. Assess* **194**, 517 (2022).

88. Da Silva, G.N. *et al.* Management systems and soil use on fractions and stocks of organic carbon and nitrogen total in Cerrado latosol. *Biosci.J* **32**, 1482-1492 (2016).

89. Da Silva, R. B. *et al.* A soil quality physical–chemical approach 30 years after land-use change from forest to banana plantation. *Environ. Monit. Assess* **194**, 482 (2022).

90. Damian, J. M. *et al.* Deforestation and land use change mediate soil carbon changes in the eastern Brazilian Amazon. *Reg. Environ. Change* **21**, 64 (2021).

91. Damian, J. M. *et al.* Predicting soil C changes after pasture intensification and diversification in Brazil. *CATENA* **202**, 105238 (2021).

92. De Abreu, L. H. G. *et al.* Variation in soil carbon, nitrogen and microbial attributes within a silvopastoral system in the Brazilian Cerrado. *Agrofor. Syst* **94**, 2343–2353 (2020).

93. De Assis, C. P., Maia, S. M. F., Nogueira, R. S., de Alencar, G. V. & de Oliveira, T. S. Soil Organic Matter Changes in Agroforestry and Organic Farming in the Semi-Arid Region of Northeastern Brazil. *Global Science Books* **5**,36-44(2011).

94. De Bona, F. D., Bayer, C., Dieckow, J. & Bergamaschi, H. Soil quality assessed by carbon management index in a subtropical Acrisol subjected to tillage systems and irrigation. *Soil Res* **46**, 469-475 (2008).

95. De Camargo, P. B. *et al.* Soil carbon dynamics in regrowing forest of eastern Amazonia. *Glob. Change Biol* **5**, 693–702 (1999).

96. De Castro Paes, É. *et al.* Land use, management and physical attributes of dense Ferralsols in tropical northeastern Brazil. *CATENA* **203**, 105321 (2021).

97. De Figueiredo, C. C. *et al.* Relationships between soil organic matter pools and nitrous oxide emissions of agroecosystems in the Brazilian Cerrado. *Sci. Total Environ* **618**, 1572–1582 (2018).

98. De Moraes Sá, J. C. *et al.* Carbon Depletion by Plowing and its Restoration by No-Till Cropping Systems in Oxisols of Subtropical and Tropical Agro-Ecoregions in Brazil. *Land Degrad. Dev* **26**, 531–543 (2015).

99. De Moraes Sá, J. C. *et al.* Soil-Specific Inventories of Landscape Carbon and Nitrogen Stocks under no-till and native vegetation to Estimate Carbon Offset in a Subtropical Ecosystem. *Soil Sci. Soc. Am. J* **77**, 2094–2110 (2013).

100. De Morais, J. R., Castilhos, R. M. V., Lacerda, C. L., Pinto, L. F. S. & Carlos, F. S. Carbon and nitrogen stocks and microbiological attributes of soil under eucalyptus cultivation in the Pampa biome of southern Brazil. *Geoderma Reg* **25**, e00392 (2021).

101. De Oliveira Ferreira, A. *et al.* Can no-till grain production restore soil organic carbon to levels natural grass in a subtropical Oxisol? *Agric. Ecosyst. Environ* **229**, 13–20 (2016).

102. De Oliveira, R. L. L. *et al.* Management Practices Affect Soil Carbon and Physical Quality in Oil Palm Agroforestry Systems in the Amazon. *J. Soil Sci. Plant Nutr* **22**, 4653–4668 (2022).

103. De Oliveira, S. P., De Lacerda, N. B., Blum, S. C., Escobar, M. E. O. & De Oliveira, T. S. Organic Carbon and Nitrogen Stocks in Soils of Northeastern Brazil Converted to Irrigated Agriculture. *Land Degrad. Dev* **26**, 9–21 (2015).

104. De Sant-Anna, S. A. C. *et al.* Changes in soil organic carbon during 22 years of pastures, cropping or integrated crop/livestock systems in the Brazilian Cerrado. *Nutr. Cycl. Agroecosystems* **108**, 101–120 (2017).

105. De Souza Medeiros, A., Malta Ferreira Maia, S., Dos Santos, T. C. & De Araújo Gomes, T. C. Soil carbon losses in conventional farming systems due to land-use change in the Brazilian semi-arid region. *Agric. Ecosyst. Environ* **287**, 106690 (2020).

106. Denardin, R. B. N. *et al.* Estoque de carbono no solo sob diferentes formações florestais, Chapecó - SC. *Ciênc. Florest* **24**, 59–69 (2014).

107. Desjardins, T., Andreux, F., Volkoff, B. & Cerri, C. C. Organic carbon and 13C contents in soils and soil size-fractions, and their changes due to deforestation and pasture installation in eastern Amazonia. *Geoderma* **61**, 103–118 (1994).

108. Dias, B. D. O., Silva, C. A., Soares, E. M. B. & Bettiol, W. Estoque de carbono e quantificação de substâncias húmicas em Latossolo submetido a aplicação contínua de lodo de esgoto. *Rev. Bras. Ciênc. Solo* **31**, 701–711 (2007).

109. Dias, F. P. M., Leandro, W. M., Fernandes, P. M. & Xavier, F. A. D. S. Impact of short-term land-use change on soil organic carbon dynamics in transitional agro-ecosystems: a case study in the Brazilian Cerrado. *Carbon Manag* **13**, 238–248 (2022).

110. Dieckow, J. et al. Land use, tillage, texture and organic matter stock and composition in tropical and subtropical Brazilian soils. *Eur. J. Soil Sci* **60**, 240–249 (2009).

111. Diekow, J. et al. Soil C and N stocks as affected by cropping systems and nitrogen fertilisation in a southern Brazil Acrisol managed under no-tillage for 17 years. *Soil Tillage Res* **81**, 87–95 (2005).

112. Diógenes, L. C. *et al.* Microbial attributes and carbon and nitrogen stocks in Latosol under monocropping and intercropping irrigated. Rev. Ciênc. Agrar. - Amaz. *J. Agric. Environ. Sci* **56**, 106–111 (2013).

113. Dionizio, E. A., Pimenta, F. M., Lima, L. B. & Costa, M. H. Carbon stocks and dynamics of different land uses on the Cerrado agricultural frontier. *PLOS ONE* **15**, e0241637 (2020).

114. Dortzbach, D., Assunção, S. A., Pereira, M. G. & Silva Neto, E. C. D. Fractions of soil organic matter in the vineyards of altitude regions in Santa Catarina. *Semina Ciênc. Agrár* **38**, 1799 (2017).

115. Dos Santos, A. P. *et al.* Nitrogen and carbon stocks in sandy soil cultivated with corn subjected to 17 years of animal manure and mineral fertilizer application. *Agric. Ecosyst. Environ* **367**, 108981 (2024).

116. Dos Santos, C. A. *et al.* Changes in soil carbon stocks after land-use change from native vegetation to pastures in the Atlantic forest region of Brazil. *Geoderma* **337**, 394–401 (2019).

117. Dos Santos, U. J. *et al.* Soil organic carbon fractions and humic substances are affected by land uses of Caatinga forest in Brazil. *Arid Land Res. Manag* **33**, 255–273 (2019).

118. Durigan, M. *et al.* Soil Organic Matter Responses to Anthropogenic Forest Disturbance and Land Use Change in the Eastern Brazilian Amazon. *Sustainability* **9**, 379 (2017).

119. Fabrizzi, K. P. *et al.* Protection of soil organic C and N in temperate and tropical soils: effect of native and agroecosystems. *Biogeochemistry* **92**, 129–143 (2009).

120. Falconeres Vogado, R. *et al.* Soil organic carbon stocks and fractions under integrated systems and pasture in the Cerrado of Northeast Brazil. *CATENA* **243**, 108196 (2024).

121. Cruvinel, Ê. B.F., Bustamante, M. M. D. C., Kozovits, A. R. & Zepp, R. G. Soil emissions of NO, N2O and CO2 from croplands in the savanna region of central Brazil. *Agric. Ecosyst. Environ* **144**, 29–40 (2011).

122. Ferraz-Almeida, R. *et al.* Sorghum–Grass Intercropping Systems under Varying Planting Densities in a Semi-Arid Region: Focusing on Soil Carbon and Grain Yield in the Conservation Systems. *Agriculture* **12**, 1762 (2022).

123. Ferreira *et al.* Organic carbon stock changes and crop yield in a tropical sandy soil under rainfed grains-cotton farming systems in Bahia, Brazil. *Pesq. Agropec. Trop.* **52**, e71219 (2022).

124. Ferreira, A. C. D. B. *et al.* Soil carbon accumulation in cotton production systems in the Brazilian Cerrado. *Acta Sci. Agron* **42**, e43039 (2019).

125. Ferreira, C. D. R. *et al.* Dynamics of soil aggregation and organic carbon fractions over 23 years of no-till management. *Soil Tillage Res* **198**, 104533 (2020).

126. Ferreira, C. D. S. *et al.* Stock of Carbon and Soil Organic Fractions in No-Tillage and Crop–Livestock Integration Systems. *Sustainability* **16**, 3025 (2024).

127. Ferreira, R. V., Tavares, R. L. M., Medeiros, S. F. D., Silva, A. G. D. & Silva Júnior, J. F. D. Carbon stock and organic fractions in soil under monoculture and Sorghum bicolor–Urochloa ruziziensis intercropping systems. *Bragantia* **79**, 425–433 (2020).

128. Ferri, M. V. W., Vidal, R. A., Gomes, J., Dick, D. P. & Souza, R. F. D. Atividade do herbicida acetochlor em solo submetido à semeadura direta e ao preparo convencional. *Pesqui. Agropecuária Bras* **37**, 1697–1703 (2002).

129. Fidalski, J. & Tormena, C. A. Physical quality of sandy soils under orange orchards in Southern Brazil. *Rev. Bras. Ciênc. Solo* **46**, e0220006 (2022).

130. Fidalski, J., Yagi, R. & Tormena, C. A. Revolvimento Ocasional e Calagem em Latossolo Muito Argiloso em Sistema Plantio Direto Consolidado. *Rev. Bras. Ciênc. Solo* **39**, 1483–1489 (2015).

131. Figueiredo, C. C., Ramos, M. L. G. & Tostes, R. Propriedades Físicas e Matéria Orgânica de um Latossolo Vermelho Sob Sistemas de Manejo e Cerrado Nativo. *Biosci J* **24**, 24-30 (2008).

132. Figueiredo, C. C., Resck, D. V. S., Carneiro, M. A. C., Ramos, M. L. G. & Sá, J. C. M. Stratification ratio of organic matter pools influenced by management systems in a weathered Oxisol from a tropical agro-ecoregion in Brazil. *Soil Res* **51**, 133-141 (2013).

133. Figueiredo, E. B. D. *et al.* Soil CO2 –C Emissions and Correlations with Soil Properties in Degraded and Managed Pastures in Southern Brazil. *Land Degrad. Dev* **28**, 1263–1273 (2017).

134. Filho, J. F. L., De Oliveira, H. M. R., De Souza Barros, V. M., Dos Santos, A. C. & De Oliveira, T. S. From forest to pastures and silvopastoral systems: Soil carbon and nitrogen stocks changes in northeast Amazônia. *Sci. Total Environ* **908**, 168251 (2024).

135. Fracetto, F. J. C., Fracetto, G. G. M., Cerri, C. C., Feigl, B. J. & Siqueira Neto, M. Estoques de carbono e nitrogênio no solo cultivado com mamona na Caatinga. *Rev. Bras. Ciênc. Solo* **36**, 1545–1552 (2012).

136. Franchini, J., Crispino, C., Souza, R., Torres, E. & Hungria, M. Microbiological parameters as indicators of soil quality under various soil management and crop rotation systems in southern Brazil. *Soil Tillage Res* **92**, 18–29 (2007).

137. Franco, A. L. C. *et al.* Soil carbon, nitrogen and phosphorus changes under sugarcane expansion in Brazil. *Sci. Total Environ* **515–516**, 30–38 (2015).

138. Frazão, L. A. *et al.* Carbon and nitrogen stocks and organic matter fractions in the topsoil of traditional and agrisilvicultural systems in the Southeast of Brazil. *Soil Res* **59**, 794–805 (2021).

139. Frazão, L. A. *et al.* Soil carbon stocks and changes after oil palm introduction in the Brazilian Amazon. *GCB Bioenergy* **5**, 384-390 (2013).

140. Frazão, L. A., Paustian, K., Cerri, C. E. P. & Cerri, C. C. Soil carbon stocks under oil palm plantations in Bahia State, Brazil. *Biomass Bioenergy* **62**, 1–7 (2014).

141. Frazão, L. A., Piccolo, M. D. C., Feigl, B. J., Cerri, C. C. & Cerri, C. E. P. Inorganic nitrogen, microbial biomass and microbial activity of a sandy Brazilian Cerrado soil under different land uses. *Agric. Ecosyst. Environ* **135**, 161–167 (2010).

142. Freitas, I. C. D. *et al.* Agrosilvopastoral Systems and Well-Managed Pastures Increase Soil Carbon Stocks in the Brazilian Cerrado. Rangel. *Ecol. Manag* **73**, 776–785 (2020).

143. Freitas, I. C. D. *et al.* Changing the land use from degraded pasture into integrated farming systems enhance soil carbon stocks in the Cerrado biome. *Acta Sci. Agron* **46**, 1-12 (2024).

144. Freitas, I. C. D. *et al.* Soil Carbon and Nitrogen Stocks under Agrosilvopastoral Systems with Different Arrangements in a Transition Area between Cerrado and Caatinga Biomes in Brazil. *Agronomy* **12**, 2926 (2022).

145. Freitas, P. L. D., Blancaneaux, P., Gavinelli, E., Larré-Larrouy, M.-C. & Feller, C. Nível e natureza do estoque orgânico de latossolos sob diferentes sistemas de uso e manejo. *Pesqui. Agropecuária Bras* **35**, 157–170 (2000).

146. Freitas, R. D. C. A. D. *et al.* Soil Organic Matter Quality in Jatropha spp. Plantations in Different Edaphoclimatic Conditions. *Rev. Bras. Ciênc. Solo* **41**, (2017).

147. Freixo, A. A., Machado, P. L. O. de A., Dos Santos, Henrique. P., Silva, C. A. & Fadigas, F. D. S. Soil organic carbon and fractions of a Rhodic Ferralsol under the influence of tillage and crop rotation systems in southern Brazil. *Soil Tillage Res* **64**, 221–230 (2002).

148. Galdos, M. V., Cerri, C. C. & Cerri, C. E. P. Soil carbon stocks under burned and unburned sugarcane in Brazil. *Geoderma* **153**, 347–352 (2009).

149. Gazolla, P. R. *et al.* Estoque de carbono e atributos físicos de um Latossolo Vermelho em diferentes sistemas de manejo. *Agra- Ver. Bras.Ciênc. Agrárias* **8**, 229-235 (2013).

150. Giácomo, R. G., Pereira, M. G., Guareschi, R. F. & Machado, D. L. Atributos Químicos e Físicos do Solo, Estoques de Carbono e Nitrogênio e Frações Húmicas em Diferentes Formações Vegetais. *Ciênc. Florest* **25**, 617–631 (2015).

151. Gmach, M.-R. *et al.* Soil organic matter dynamics and land-use change on Oxisols in the Cerrado, Brazil. *Geoderma Reg* **14**, e00178 (2018).

152. Gualberto, A. V. S. *et al.* Organic C Fractions in Topsoil under Different Management Systems in Northeastern Brazil. *Soil Syst* **7**, 11 (2023).

153. Guareschi, R. F. *et al.* Estoque de carbono em Latossolo Vermelho Distroférrico sob diferentes sistemas de manejo. *Rev. Bras. de Ciênc. Agrárias* **7**, 597-602 (2012).

154. Guareschi, R. F., Pereira, M. G. & Perin, A. Frações da matéria orgânica em áreas de Latossolo sob diferentes sistemas de manejo no Cerrado do estado de Goiás. *Semina Ciênc. Agrár* **34**, 2615 (2013).

155. Guera, K. C. S., Fonseca, A. F. D. & Ribeiro, F. Stocks and Distribution of Soil Carbon, Nitrogen, Phosphorus and Sulfur in an Integrated Crop-Livestock System Treated with Phosphates. *Braz. Arch. Biol. Technol* **63**, e20190520 (2020).

156. Guerrini, I. A. *et al.* Evaluating carbon stocks in soils of fragmented Brazilian Atlantic Forests (BAF) based on soil features and different methodologies. *Sci. Rep* **14**, 10007 (2024).

157. Guimarães, M. F. *et al.* Monitoring changes in the chemical properties of an oxisol under long-term no-tillage management in subtropical Brazil. *Soil Sci* **173**, 408–416 (2008).

158. Hickmann, C. & Costa, L. M. D. Estoque de carbono no solo e agregados em Argissolo sob diferentes manejos de longa duração. *Rev. Bras. Eng. Agríc. E Ambient* **16**, 1055–1061 (2012).

159. Hughes, R. F., Kauffman, J. B. & Cummings, D. L. Dynamics of Aboveground and Soil Carbon and Nitrogen Stocks and Cycling of Available Nitrogen along a Land-use Gradient in Rondônia, Brazil. *Ecosystems* **5**, 244–259 (2002).

160. Isernhagen, E. C. C. *et al.* Estoques de carbono lábil e total em solo sob integração lavoura-pecuária-floresta na região de transição Cerrado/Amazônia. *Nativa Agric. Envir. Resear* **5**, 515-521 (2017).

161. Jantalia, C. P. *et al.* Tillage effect on C stocks of a clayey Oxisol under a soybean-based crop rotation in the Brazilian Cerrado region. *Soil Tillage Res* **95**, 97–109 (2007).

162. Junior, C. C. *et al.* Carbono total e δ13C em agregados do solo sob vegetação nativa e pastagem no bioma cerrado. *Rev. Bras. Ciênc. Solo* **35**, 1241-1252 (2011).

163. Junior, P. R. D. R. *et al.* Soil carbon stock in silvopastoral system, pasture and sugarcane culture. *Idesia Arica* **32**, 35–42 (2014).

164. Kato, E. Propriedades físicas e teor de carbono orgânico de um latossolo vermelho-amarelo do cerrado, sob diferentes coberturas vegetais. *Bioscience J* **25**, 732–738 (2010).

165. Koutika, L.-S. *et al.* Organic matter dynamics and aggregation in soils under rain forest and pastures of increasing age in the eastern Amazon Basin. *Geoderma* **76**, 87–112 (1997).

166. Kuneski, A. C. *et al.* Effects of Tillage and Cover Crops on Total Carbon and Nitrogen Stocks and Particle-Size Fractions of Soil Organic Matter under Onion Crop. *Horticulturae* **9**, 822 (2023).

167. Lammel, D. R. *et al.* C and N stocks are not impacted by land use change from Brazilian Savanna (Cerrado) to agriculture despite changes in soil fertility and microbial abundances. *J. Plant Nutr. Soil Sci* **180**, 436–445 (2017).

168. Leite, L. F. C. Soil organic carbon and biological indicators in an acrisol under tillage systems and organic management in north-eastern Brazil. Australian *J. Soil Res* **48**, 258-265 (2010).

169. Leite, L. F. C., Galvão, S. R. S., Holanda Neto, M. R., Araújo, F. S. & Iwata, B. F. Atributos químicos e estoques de carbono em Latossolo sob plantio direto no cerrado do Piauí. *Rev. Bras. Eng. Agríc. E Ambient* **14**, 1273–1280 (2010).

170. Leite, L. F. C., Iwata, B. D. F. & Araújo, A. S. F. Soil organic matter pools in a tropical savanna under agroforestry system in Northeastern Brazil. *Rev. Árvore* **38**, 711–723 (2014).

171. Leite, L. F. C., Mendonça, E. S., Machado, P. L. O. A. & Matos, E. S. Total C and N storage and organic C pools of a Red-Yellow Podzolic under conventional and no tillage at the Atlantic Forest Zone, south-eastern Brazil. *Soil Res* **41**, 717 (2003).

172. Lemos, E. C. M., Vasconcelos, S. S., Santiago, W. R., De Oliveira Junior, M. C. M. & De A. Souza, C. M. The responses of soil, litter and root carbon stocks to the conversion of forest regrowth to crop and tree production systems used by smallholder farmers in eastern Amazonia. *Soil Use Manag* **32**, 504–514 (2016).

173. Lima, A. F. L. D. *et al.* The Stability of Aggregates in Different Amazonian Agroecosystems Is Influenced by the Texture, Acidity, and Availability of Ca and Mg in the Soil. *Agronomy* **14**, 677 (2024).

174. Lima, A. M. N. *et al.* Frações da matéria orgânica do solo após três décadas de cultivo de eucalipto no Vale do Rio Doce-MG. *Rev. Bras. Ciênc. Solo* **32**, 1053–1063 (2008).

175. Lima, C. *et al.* Forest- and pasture-derived carbon contributions to carbon stocks and microbial respiration of tropical pasture soils. *Oecologia* **107**, 113–119 (1996).

176. Lima, C. L. R. D., Dupont, P. B., Pillon, C. N. & Miola, E. C. C. Least limiting water range, S-index and compressibility of a Udalf under different management systems. *Sci. Agric* **77**, e20170224 (2020).

177. Lima, D. T. D. *et al.* Organic carbon and carbon stock: relations with physical indicators and soil aggregation in areas cultivated with sugar cane. *Trop. Subtrop. Agroecosystems* **20**, 341 - 352 (2017).

178. Lira Junior, M. A., Fracetto, F. J. C., Ferreira, J. D. S., Silva, M. B. & Fracetto, G. G. M. Legume-based silvopastoral systems drive C and N soil stocks in a subhumid tropical environment. *CATENA* **189**, 104508 (2020).

179. Locatelli, J. L. *et al.* Soil Strength and Structural Stability Are Mediated by Soil Organic Matter Composition in Agricultural Expansion Areas of the Brazilian Cerrado Biome. *Agronomy* **13**, 71 (2022).

180. Locatelli, J.L. *et al.* Soil carbon sequestration and stocks: short-term impact of maize succession to cover crops in Southern Brazil Inceptisol. *Span. J. Agric. Res* **18**, e0304(2020).

181. Lopes, E. L. N., Fernandes, A. R., Teixeira, R. A., Sousa, E. S. D. & Ruivo, M. D. L. P. Soil attributes under different crop management systems in an Amazon Oxisols. *Bragantia* **74**, 428–435 (2015).

182. Loss, A. *et al.* Oxidizable carbon and humic substances in rotation systems with brachiaria/livestock and pearl millet/no livestock in the Brazilian Cerrado. *Span. J. Agric. Res* **11**, 217-231 (2013).

183. Loss, A., Pereira, M. G., Mendes Costa, E., Beutler, S. J. & De Cássia Piccolo, M. Soil fertility, humic fractions and natural abundance of 13C and 15N in soil under different land use in Paraná State, Southern Brazil. *Idesia Arica* **34**, 27–38 (2016).

184. Loss, A., Ribeiro, E. C., Pereira, M. G. & Costa, E. M. Atributos físicos e químicos do solo em sistemas de consórcio e sucessão de lavoura, pastagem e silvipastoril em Santa Teresa, ES. *Biosci J* **30**, 1347-1357 (2014).

185. Lovato, T., Mielniczuk, J., Bayer, C. & Vezzani, F. Adição de carbono e nitrogênio e sua relação com os estoques no solo e com o rendimento do milho em sistemas de manejo. *Rev. Bras. Ciênc.* Solo **28**, 175–187 (2004).

186. Luca, E. F. D. *et al.* Avaliação de atributos físicos e estoques de carbono e nitrogênio em solos com queima e sem queima de canavial. *Rev. Bras. Ciênc. Solo* **32**, 789–800 (2008).

187. Luca, E. F. *et al.* Effect of conversion from sugarcane preharvest burning to residues green-trashing on SOC stocks and soil fertility status: Results from different soil conditions in Brazil. *Geoderma* **310**, 238–248 (2018).

188. Macedo, M. C. M. Integração lavoura e pecuária: o estado da arte e inovações tecnológicas. *Rev. Bras. Zootec* **38**, 133–146 (2009).

189. Macedo, R. S. *et al.* Agroforestry can improve soil fertility and aggregate-associated carbon in highland soils in the Brazilian northeast. *Agrofor. Syst* **98**, 1167–1179 (2024).

190. Machado, J. D. S., Oliveira Filho, L. C. I., Santos, J. C. P., Paulino, A. T. & Baretta, D. Morphological diversity of springtails (Hexapoda: Collembola) as soil quality bioindicators in land use systems. *Biota Neotropica* **19**, e20180618 (2019).

191. Machado, P. L. O. A, Silva, C.A. Soil management under no-tillage systems in the tropics with special reference to Brazil. *Nutrient Cycling in Agroecosystems* **61**, 119-130 (2001).

192. Machado, P. L. O. A. et al. Carbon stocks of a Rhodic Ferralsol under no-tillage in Southern Brazil: spatial variability at a farm scale. *Soil Res* **47**, 253 (2009).

193. Magalhaes, S. S. D. A., Ramos, F. T. & Weber, O. L. D. S. Carbon stocks of an Oxisol after thirty-eight years under different tillage systems. *Rev. Bras. Eng. Agríc. E Ambient* **20**, 85–91 (2016).

194. Maia, S. M. F., Ogle, S. M., Cerri, C. C. & Cerri, C. E. P. Changes in soil organic carbon storage under different agricultural management systems in the Southwest Amazon Region of Brazil. *Soil Tillage Res* **106**, 177–184 (2010).

195. Maia, S. M. F., Ogle, S. M., Cerri, C. E. P. & Cerri, C. C. Effect of grassland management on soil carbon sequestration in Rondônia and Mato Grosso states, Brazil. *Geoderma* **149**, 84–91 (2009).

196. Maia, S. M. F., Otutumi, A. T., Mendonça, E. D. S., Neves, J. C. L. & Oliveira, T. S. D. Combined effect of intercropping and minimum tillage on soil carbon sequestration and organic matter pools in the semiarid region of Brazil. *Soil Res* **57**, 266 (2019).

197. Maia, S.M.F. *et al.* Organic carbon pools in a Luvisol under agroforestry and conventional farming systems in the semi-arid. *Agroforestry Systems* **71**, 127-138 (2007).

198. Maltas, A. *et al.* Long-term effects of continuous direct seeding mulch-based cropping systems on soil nitrogen supply in the Cerrado region of Brazil. *Plant Soil* **298**, 161–173 (2007).

199. Mantovanelli, B. C. *et al.* Avaliação dos atributos do solo sob diferentes usos na região de Humaitá, Amazonas. *Rev. Cienc. Agrar* **58**, 122-130 (2015).

200. Maquere, V. *et al.* Influence of land use (savanna, pasture, Eucalyptus plantations) on soil carbon and nitrogen stocks in Brazil. *Eur. J. Soil Sci* **59**, 863–877 (2008).

201. Marafon, G. *et al.* C and P pool restoration by a no-tillage system on Brazilian Cerrado Oxisol in Piauí State. *Environ. Monit. Assess* **192**, 254 (2020).

202. Marchão, R. L. *et al.* Carbon and nitrogen stocks in a Brazilian clayey Oxisol: 13-year effects of integrated crop–livestock management systems. *Soil Tillage Res* **103**, 442–450 (2009).

203. Marinho Junior, J. L. *et al.* Carbon Dynamics in Humic Fractions of Soil Organic Matter Under Different Vegetation Cover in Southern Tocantins. *Floresta E Ambiente* **28**, e20200024 (2021).

204. Monroe, P. H.M., Bittencourt Barreto-Garcia, P. A., Barros, W. T., Romeiro Barbosa De Oliveira, F. G. & Pereira, M. G. Physical protection of soil organic carbon through aggregates in different land use systems in the semi-arid region of Brazil. *J. Arid Environ* **186**, 104427 (2021).

205. Martíni, A. F. *et al.* Soil physical quality response to management systems in a long-term sugarcane trial. *Land Degrad. Dev* **35**, 1320–1334 (2024).

206. Martins, A. P. *et al.* Short-term Impacts on Soil-quality Assessment in Alternative Land Uses of Traditional Paddy Fields in Southern Brazil. *Land Degrad. Dev* **28**, 534–542 (2017).

207. Martins, C. M., Galindo, I. C. D. L., Souza, E. R. D. & Poroca, H. A. Atributos químicos e microbianos do solo de áreas em processo de desertificação no semiárido de Pernambuco. *Rev. Bras. Ciênc. Solo* **34**, 1883–1890 (2010).

208. Martins, L. F.B. N. et al. Soil carbon stock in different uses in the southern cone of Mato Grosso do Sul. *Rev. de Agric. Neotropical* **7**, 86-94 (2020).

209. Matias, M. D. C. B. D. S., Salviano, A. A. C., Leite, L. F. D. C. & Araújo, A. S. F. D. Biomassa microbiana e estoques de C e N do solo em diferentes sistemas de manejo, no Cerrado do Estado do Piauí. *Acta Sci. Agron* **31**, 517–521 (2009).

210. Matos, P. S. *et al.* Linkages among Soil Properties and Litter Quality in Agroforestry Systems of Southeastern Brazil. *Sustainability* **12**, 9752 (2020).

211. Matos, P. S. et al. Soil organic carbon fractions in agroforestry system in Brazil: seasonality and short-term dynamic assessment. *Rev. Bras. Ciênc. Solo* **47**, e0220095 (2023).

212. Mattei, E. *et al.* Carbon, nitrogen, and organic matter of soil in an integrated crop-livestock system. *Rev. Agric. Neotropical* **7**, 7-14 (2020).

213. Medeiros, A. D. S., Soares, A. A. S. & Maia, S. M. F. Soil carbon stocks and compartments of organic matter under conventional systems in Brazilian semi-arid region. *Rev. Caatinga* **35**, 697–710 (2022).

214. Mello, F. F. C. *et al.* Payback time for soil carbon and sugar-cane ethanol. *Nat. Clim. Change* **4**, 605–609 (2014).

215. Mello, J. M. *et al.* Dinâmica dos Atributos Físico-Químicos e Variação Sazonal dos Estoques de Carbono no Solo em Diferentes Fitofisionomias do Pantanal Norte Mato-Grossense. *Rev. Árvore* **39**, 325–336 (2015).

216. Melo, D. M. A. D. *et al.* Soil Quality Indicators in Peasant Agroecosystems in Paraíba State, Brazil. *Trop. Conserv. Sci* **17**, 19400829231215492 (2024).

217. Melo, G. B., Pereira, M. G., Perin, A., Guareschi, R. F. & Soares, P. F. C. Estoques e frações da matéria orgânica do solo sob os sistemas plantio direto e convencional de repolho. *Pesqui. Agropecuária Bras* **51**, 1511–1519 (2016).

218. Metay, A. *et al.* Storage and forms of organic carbon in a no-tillage under cover crops system on clayey Oxisol in dryland rice production (Cerrados, Brazil). *Soil Tillage Res* **94**, 122–132 (2007).

219. Monroe, P. H. M., Gama-Rodrigues, E. F., Gama-Rodrigues, A. C. & Marques, J. R. B. Soil carbon stocks and origin under different cacao agroforestry systems in Southern Bahia, Brazil. *Agric. Ecosyst. Environ 221*, 99–108 (2016).

220. Moraes, J. F. L. *et al.* Soil properties under Amazon Forest and changes due to pasture installation in Rondônia, Brazil. *Geoderma* **70**,63-81(1996).

221. Morais, V. A. *et al.* Spatial and vertical distribution of litter and belowground carbon in a Brazilian Cerrado vegetation. *CERNE* **23**, 43–52 (2017).

222. Mota, P. K. *et al.* Soil physical quality in response to intensification of grain production systems. *Rev. Bras. Eng. Agríc. E Ambient* **24**, 647–655 (2020).

223. Muller, M. M. L. & Desjardins, T. Degradação de pastagens na Região Amazônica: propriedades físicas do solo e crescimento de raízes. *Pesq. agropec. Bras., Brasília* **36**, 1409-1418 (2001).

224. Nascimento, P. C. D. *et al.* Sistemas de manejo e a matéria orgânica de solo de várzea com cultivo de arroz. *Rev. Bras. Ciênc. Solo* **33**, 1821–1827 (2009).

225. Neill, C. *et al.* Soil carbon and nitrogen stocks following forest clearing for pasture in the Southwestern Brazilian Amazon. *Ecol. Appl* **7**, 1216–1225 (1997).

226. Neto, J. F. *et al.* Soil carbon and nitrogen fractions and physical attributes affected by soil acidity amendments under no-till on Oxisol in Brazil. *Geoderma Regional* **24**, e00347 (2021).

227. Neto, M. S. *et al.* Soil carbon stocks under no-tillage mulch-based cropping systems in the Brazilian Cerrado: An on-farm synchronic assessment. *Soil Tillage Res* **110**, 187–195 (2010).

228. Oliveira Filho, J. D. S. *et al.* Assessing the effects of 17 years of grazing exclusion in degraded semi-arid soils: Evaluation of soil fertility, nutrients pools and stoichiometry. *J. Arid Environ* **166**, 1–10 (2019).

229. Oliveira, F. É. R. D., Oliveira, J. D. M. & Xavier, F. A. D. S. Changes in Soil Organic Carbon Fractions in Response to Cover Crops in an Orange Orchard. *Rev. Bras. Ciênc. Solo* **40**, (2016).

230. Oliveira, I. N. D. *et al.* Tillage systems impact on soil physical attributes, sugarcane yield and root system propagated by pre-sprouted seedlings. *Soil Tillage Res* **223**, 105460 (2022).

231. Oliveira, J. D. M. *et al.* Integrated farming systems for improving soil carbon balance in the southern Amazon of Brazil. *Reg. Environ. Change* **18**, 105–116 (2018).

232. Oliveira, J. F., De Oliveira, J. C. S., Ruiz, D. B., De Cesare Barbosa, G. M. & Filho, J. T. Changes in carbon and phosphorus storages and humic substances in a Ferralsol, after tillage and animal manures applications. *Soil Tillage Res* **220**, 105358 (2022).

233. Oliveira, J. M. *et al.* Predicting Soil Organic Carbon Dynamics of Integrated Crop-Livestock System in Brazil Using the CQESTR Model. *Front. Environ. Sci* **10**, 826786 (2022).

234. Oliveira, P. P. A. *et al.* Potential of integrated trees-pasture-based systems for GHG emission mitigation and improving soil carbon dynamics in the Atlantic Forest biome, Southeastern of Brazil. *Eur. J. Agron* **158**, 127219 (2024).

235. Paiva, A. O., Rezende, A. V. & Pereira, R. S. Estoque de carbono em cerrado sensu stricto do Distrito Federal. *Rev. Árvore* **35**, 527–538 (2011).

236. Pedra, W.S. *et al.* Carbon and nitrogen stocks under different management conditions of a yellow-red ultisol, cultivated with sweet corn in Sergipe coastal tablelands. *Semina: Ciênc Agrá* **33**, 2075-2090 (2012).

237. Pegoraro, R. F. *et al.* Estoques de carbono e nitrogênio nas frações da matéria orgânica em argissolo sob eucalipto e pastagem. *Ciênc. Florest* **21**, 261–273 (2011).

238. Pereira, F. D. S. *et al.* Qualidade física de um Latossolo Vermelho submetido a sistemas de manejo avaliado pelo Índice S. *Rev. Bras. Ciênc. Solo* **35**, 87–95 (2011).

239. Pereira, M. G., Loss, A., Beutler, S. J. & Torres, J. L. R. Carbono, matéria orgânica leve e fósforo remanescente em diferentes sistemas de manejo do solo. *Pesqui. Agropecuária Bras* **45**, 508–514 (2010).

240. Pessôa, G. C. M. *et al.* Carbon and Nitrogen Stocks and Microbiological Activity Under Forest-Pasture System and Traditional Pasture in Pernambuco. Floresta E Ambiente 29, e20210068 (2022).

241. Petter, F. A. *et al.* Carbon stocks in oxisols under agriculture and forest in the southern Amazon of Brazil. *Geoderma Reg* **11**, 53–61 (2017).

242. Pinheiro, É. F. M., De Campos, D. V. B., De Carvalho Balieiro, F., Dos Anjos, L. H. C. & Pereira, M. G. Tillage systems effects on soil carbon stock and physical fractions of soil organic matter. *Agric. Syst* **132**, 35–39 (2015).

243. Pinheiro, E.F.M. *et al.* Impact of pre-harvest burning versus trash conservation on soil carbon and nitrogen stocks on a sugarcane plantation in the Brazilian Atlantic Forest region. *Plant and Soil* **333**,71-80(2010).

244. Piva, J. T. *et al.* No-tillage and crop-livestock with silage production impact little on carbon and nitrogen in the short-term in a subtropical Ferralsol. *Rev. Braz. J. Agric. Sci* **15**, 1–7 (2020).

245. Portella, C. M. R., Guimarães, M. D. F., Feller, C., Fonseca, I. C. D. B. & Tavares Filho, J. Soil aggregation under different management systems. *Rev. Bras. Ciênc. Solo* **36**, 1868–1877 (2012).

246. Potes, M. D. L., Dick, D. P., Santana, G. S., Tomazi, M. & Bayer, C. Soil organic matter in fire-affected pastures and in an Araucaria forest in South-Brazilian Leptosols. *Pesqui. Agropecuária Bras* **47**, 707–715 (2012).

247. Pulrolnik, K., Barros, N. F. D., Silva, I. R., Novais, R. F. & Brandani, C. B. Estoques de carbono e nitrogênio em frações lábeis e estáveis da matéria orgânica de solos sob eucalipto, pastagem e cerrado no Vale do Jequitinhonha - MG. *Rev. Bras. Ciênc. Solo* **33**, 1125–1136 (2009).

248. Quartucci, F., Gocke, M., Denich, M., De Moraes Gonçalves, J. L. & Amelung, W. Deep soil carbon loss offsets rapid aboveground carbon accumulation after reforestation. *For. Ecol. Manag* **548**, 121403 (2023).

249. Rachid, C. T. *et al*. Physical-chemical and microbiological changes in Cerrado Soil under differing sugarcane harvest management systems. *BMC Microbiol* **12**, 170 (2012).

250. Ramalho, B. *et al*. No-tillage and ryegrass grazing effects on stocks, stratification and lability of carbon and nitrogen in a subtropical Umbric Ferralsol. *Eur. J. Soil Sci* **71**, 1106–1119 (2020).

251. Ramos, D.D. *et al*. Stocks of carbon, total nitrogen and humic substances in soil under different cropping systems. *Semina: Ciênc. Agrá* **34**, 2219–2228 (2013).

252. Rangel, O. J. P. & Silva, C. A. Estoques de carbono e nitrogênio e frações orgânicas de Latossolo submetido a diferentes sistemas de uso e manejo. *Ver. Bras. Ciênc. Solo* **31**, 1609-1623 (2007).

253. Rangel-Vasconcelos, L. G. T., Kato, O. R. & Vasconcelos, S. S. Matéria orgânica leve do solo em sistema agroflorestal de corte e trituração sob manejo de capoeira. *Pesqui. Agropecuária Bras* **47**, 1142–1149 (2012).

254. Rauber, L. P. *et al*. Physical properties and organic carbon content of a Rhodic Kandiudox fertilized with pig slurry and poultry litter. *Rev. Bras. Ciênc. Solo* **36**, 1323–1332 (2012).

255. Rego, C. A. R. M. *et al*. Chemical properties and physical fractions of organic matter in oxisols under integrated agricultural production systems. *Rev. Agric. Neotropical* **7**, 81-89 (2020).

256. Reis, C. E. S. D., Dick, D. P., Caldas, J. D. S. & Bayer, C. Carbon sequestration in clay and silt fractions of Brazilian soils under conventional and no-tillage systems. *Sci. Agric* **71**, 292–301 (2014).

257. Reis, D. A., Lima, C. L. R. D. & Bamberg, A. L. Qualidade física e frações da matéria orgânica de um Planossolo sob sistema plantio direto. Pesqui. *Agropecuária Bras* **51**, 1623–1632 (2016).

258. Resende, T. M. *et al*. Dynamics of soil organic matter in a cultivated chronosequence in the Cerrado (Minas Gerais, Brazil). *Soil Res* **55**, 750 (2017).

259. Ribeiro Mauri, L. V., Mendonça, E. D. S., Bolzan, L. J. & Angeletti, M. D. P. Olericulture No-Till System at Mountain Region: Physical and Biological Attributes of the Soil. *Appl. Environ. Soil Sci* **2024**, 1–11 (2024).

260. Ribeiro, D. O. *et al*. Carbon stocks and lability in land use and management systems in southwestern Goiás, Brazil. Pesqui. Agropecuária Trop. **53**, e74416 (2023).

261. Ribeiro, D. O., Castoldi, G., Freiberger, M. B., Silva, M. A. S. D. & Rodrigues, C. R. Physical fractionation and carbon and nitrogen stocks in soil after poultry waste applications. *Rev. Caatinga* **35**, 667–676 (2022).

262. Ribeiro, J. M. *et al*. Agrosilvopastoral system as a potential model for increasing soil carbon stocks: a century model approach. *Rev. Bras. Ciênc. Solo* **47**, e0220136 (2023).

263. Rigon, J. P. G., Calonego, J. C., Capuani, S. & Franzluebbers, A. J. Soil organic C affected by dry-season management of no-till soybean crop rotations in the tropics. *Plant Soil* **462**, 577–590 (2021).

264. Rittl, T. F., Oliveira, D. & Cerri, C. E. P. Soil carbon stock changes under different land uses in the Amazon. *Geoderma Reg* **10**, 138–143 (2017).

265. Robinson, S. J. B., Van Den Berg, E., Meirelles, G. S. & Ostle, N. Factors influencing early secondary succession and ecosystem carbon stocks in Brazilian Atlantic Forest. *Biodivers. Conserv* **24**, 2273–2291 (2015).

266. Rocha, G. P. *et al.* Caracterização e estoques de carbono de sistemas agroflorestais no Cerrado de Minas Gerais. *Ciênc. Rural* **44**, 1197–1203 (2014).

267. Romaniw, J. *et al.* Carbon dynamics in no-till soil due to the use of industrial organic waste and mineral fertilizer. Rev. Ciênc. *AGRONÔMICA* **46**, (2015).

268. Rosa, C. M. D., Castilhos, R. M. V., Pauletto, E. A., Pillon, C. N. & Leal, O. D. A. Conteúdo de carbono orgânico em planossolo háplico sob sistemas de manejo do arroz irrigado. *Rev. Bras. Ciênc. Solo* **35**, 1769–1776 (2011).

269. Rosa, M. G. D., Santos, J. C. P., Brescovit, A. D., Mafra, Á. L. & Baretta, D. Spiders (Arachnida: Araneae) in Agricultural Land Use Systems in Subtropical Environments. *Rev. Bras. Ciênc. Solo* **42**, e0160576 (2018).

270. Roscoe, R. Buurman, P. Velthorst, E. & Vasconcellos, C.A. Soil organic matter dynamics in density and particle-size fractions as revealed by the 13C/13C isotopic ratio in a Cerrado Oxisol. *Soil and Sedim* **10**, 237-240 (2000).

271. Rosset, J. S. *et al.* Carbon stock, chemical and physical properties of soils under management systems with different deployment times in western region of Paraná, Brazil. *Semina: Ciênc. Agrá* **35**, 3053–3072 (2014).

272. Rosset, J.S. Schiavo, J.A. & Atanázio, R.A.R. Atributos químicos, estoque de carbono orgânico total e das frações humificadas da matéria orgânica do solo em diferentes sistemas de manejo de cana-de-açúcar. *Semina: Ciênc. Agrá* **35**, 2351–2366(2014).

273. Rossetti, K. D. V. & Centurion, J. F. Estoque de carbono e atributos físicos de um Latossolo em cronossequência sob diferentes manejos. *Rev. Bras. Eng. Agríc. E Ambient* **19**, 252–258 (2015).

274. Rossi, C. Q. *et al.* Changes in soil C and N distribution assessed by natural δ13C and δ15N abundance in a chronosequence of sugarcane crops managed with pre-harvest burning in a Cerrado area of Goiás, Brazil. *Agric. Ecosyst. Environ* **170**, 36–44 (2013).

275. Roters, D. F. *et al.* Carbon increase and soil physical improvement in an onion no-tillage system. *Rev. Bras. Ciênc. Agrár* **16**, 1–8 (2021).

276. Sá, J. C. D. M. *et al.* Nitrogen dynamics in soil management systems. I - flux of inorganic nitrogen (NH^{4+} and NO3-). *Rev. Bras. Ciênc. Solo* **35**, 1641–1649 (2011).

277. Sá, J. C. D. M. *et al.* Organic Matter Dynamics and Carbon Sequestration Rates for a Tillage Chronosequence in a Brazilian Oxisol. *Soil Sci. Soc. Am. J* **65**, 1486–1499 (2001).

278. Sales, A. *et al.* Carbono orgânico e atributos físicos do solo sob manejo agropecuário sustentável na Amazônia legal. *Agrariae* **14**, 1-15 (2018).

279. Sales, G. D. B. *et al.* Litterfall dynamics and soil carbon and nitrogen stocks in the Brazilian palm swamp ecosystems. *For. Ecosyst* **7**, 39 (2020).

280. Salton, J. C. *et al.* Agregação e estabilidade de agregados do solo em sistemas agropecuários em Mato Grosso do Sul. *Rev. Bras. Ciênc Solo* **32**, 11–21 (2008).

281. Salton, J. C. *et al.* Teor e dinâmica do carbono no solo em sistemas de integração lavoura-pecuária. *Pesqui. Agropecuária Bras* **46**, 1349–1356 (2011).

282. Sanches, A. C., Silva, A. P., Tormena, C. A. & Rigolin, A. T. Impacto do cultivo de citros em propriedades químicas, densidade do solo e atividade microbiana de um Podzólico Vermelho-Amarelo. *Rev. Bras. Ciênc. Solo* **23**, 91–99 (1999).

283. Santana, M. D. S. *et al.* Carbon and nitrogen stocks of soils under different land uses in Pernambuco state, Brazil. *Geoderma Reg* **16**, e00205 (2019).

284. Santos, N. Z. D. *et al.* Forages, cover crops and related shoot and root additions in no-till rotations to C sequestration in a subtropical Ferralsol. *Soil Tillage Res* **111**, 208–218 (2011).

285. Santos, R. S. *et al.* Simulating soil C dynamics under intensive agricultural systems and climate change scenarios in the Matopiba region, Brazil. *J. Environ. Manage* **347**, 119149 (2023).

286. Santos, W. R. D. *et al.* Carbon and nutrient dynamics in landscapes under different levels of anthropogenic intervention in the semi-arid region of Brazil. *Agric. Ecosyst. Environ* **368**, 109020 (2024).

287. Sarto, M. V. M. *et al.* Soil microbial community and activity in a tropical integrated crop-livestock system. *Appl. Soil Ecol* **145**, 103350 (2020).

288. Sausen, T. L. *et al.* Clay content drives carbon stocks in soils under a plantation of Eucalyptus saligna Labill. in southern Brazil. *Acta Bot. Bras* **28**, 266–273 (2014).

289. Scarpare, F. V. *et al.* Tillage effects on soil physical condition and root growth associated with sugarcane water availability. *Soil Tillage Res* **187**, 110–118 (2019).

290. Scheer, M. B., Curcio, G. R. & Roderjan, C. V. Funcionalidades ambientais de solos alto montanos na Serra da Igreja, Paraná. *Rev. Bras. Ciênc. Solo* **35**, 1013–1026 (2011).

291. Schiavo, J. A., Rosset, J. S., Pereira, M. G. & Salton, J. C. Índice de manejo de carbono e atributos químicos de Latossolo Vermelho sob diferentes sistemas de manejo. *Pesqui. Agropecuária Bras* **46**, 1332–1338 (2011).

292. Seben Junior, G. D. F., Corá, J. E. & Lal, R. The effects of land use and soil management on the physical properties of an Oxisol in Southeast Brazil. *Rev. Bras. Ciênc. Solo* **38**, 1245–1255 (2014).

293. Seben Junior, G. D. F., Kuhnen, F., Sylvestre, T. D. B., Almeida, C. X. D. & Centurion, J. F. Agregação de um argissolo sob pomar de goiabeiras após aplicação de resíduos da indústria processadora de goiaba. Rev. Bras. Frutic 33, 1275–1282 (2011).

294. Segnini, A. *et al.* Carbon stock and humification index of organic matter affected by sugarcane straw and soil management. *Sci. Agric.* **70**, 321–326 (2013).

295. Segnini, A. *et al.* Soil carbon stock and humification in pastures under different levels of intensification in Brazil. *Sci. Agric* **76**, 33–40 (2019).

296. Seó, H. L. S., Machado Filho, L. C. P. & Brugnara, D. Rationally Managed Pastures Stock More Carbon than No-Tillage Fields. *Front. Environ. Sci* **5**, 87 (2017).

297. Silva, B. D. O. *et al.* Implications of converting native forest areas to agricultural systems on the dynamics of CO2 emission and carbon stock in a Cerrado soil, Brazil. *J. Environ. Manage* **358**, 120796 (2024).

298. Silva, E. F. D. *et al.* Frações lábeis e recalcitrantes da matéria orgânica em solos sob integração lavoura-pecuária. *Pesqui. Agropecuária Bras* **46**, 1321–1331 (2011).

299. Silva, F. L., Silva, M. H. M. E., S. Oliveira-Júnior, E., López-Alonso, M. & Pierangeli, M. A. P. Fertility and carbon stock in pasture and forest environments in the Southern Amazon. *Rev. Bras. Eng. Agríc. E Ambient* **28**, e270888 (2024).

300. Silva, L.F.D. *et al.* Variabilidade espacial de agregados e estoque de carbono em solos antropogênicos sob floresta nativa. *Nativa* **7**, 540–547 (2017).

301. Silva, M. A. S. D. *et al.* Propriedades físicas e teor de carbono orgânico de um Argissolo Vermelho sob distintos sistemas de uso e manejo. *Rev. Bras. Ciênc. Solo* **30**, 329–337 (2006).

302. Silva, R. F. D. *et al.* Impacts of land-use and management systems on organic carbon and water-physical properties of a Latossolo Amarelo (Oxisol). *Semina Ciênc. Agrár* **38**, 109 (2017).

303. Silva, R. F. D., Borges, C. D., Garib, D. M. & Mercante, F. M. Atributos físicos e teor de matéria orgânica na camada superficial de um argissolo vermelho cultivado com mandioca sob diferentes manejos. *Rev. Bras. Ciênc. Solo* **32**, 2435–2441 (2008).

304. Siqueira Neto, M., Venzke Filho, S. D. P., Piccolo, M. D. C., Cerri, C. E. P. & Cerri, C. C. Rotação de culturas no sistema plantio direto em Tibagi (PR): I - Sequestro de carbono no solo. *Rev. Bras. Ciênc Solo* **33**, 1013–1022 (2009).

305. Siqueira-Neto, M. *et al.* Impacts of land use and cropland management on soil organic matter and greenhouse gas emissions in the Brazilian Cerrado. *Eur. J. Soil Sci* **72**, 1431–1446 (2021).

306. Sisti, C. P. J. *et al.* Change in carbon and nitrogen stocks in soil under 13 years of conventional or zero tillage in southern Brazil. *Soil Tillage Res* **76**, 39–58 (2004).

307. Smith, C. K., De Assis Oliveira, F., Gholz, H. L. & Baima, A. Soil carbon stocks after forest conversion to tree plantations in lowland Amazonia, Brazil. *For. Ecol. Manag* **164**, 257–263 (2002).

308. Soares, M. B. *et al.* Integrated production systems: An alternative to soil chemical quality restoration in the Cerrado-Amazon ecotone. *CATENA* **185**, 104279 (2020).

309. Sommer, R., Denich, M. & Vlek, P. L. G. Carbon storage and root penetration in deep soils under small-farmer land-use systems in the Eastern Amazon region, Brazil. *Plant and Soil* **219**, 231–241 (2000).

310. Sousa Junior, J.G.A. *et al.* Three-Year Soil Carbon and Nitrogen Responses to Sugarcane Straw Management. BioEnergy Res. 11, 249-261 (2018).

311. Souza Neto, E.L. *et al.* Physical quality of an Oxisol under an integrated crop-livestock-forest system in the Brazilian Cerrado. *R. Bras. Ci. Solo* **38**, 608-618 (2014).

312. Souza Nunes, R. D., Castro Lopes, A. A. D., Sousa, D. M. G. D. & Carvalho Mendes, I. D. Sistemas de manejo e os estoques de carbono e nitrogênio em latossolo de cerrado com a sucessão soja-milho. *Rev. Bras. Ciênc. Solo* **35**, 1407–1419 (2011).

313. Souza, B. V. D., Souto, P. C., Souto, J. S., Sales, F. D. C. V. & Souza Junior, C. M. P. D. Carbon in soil in different phisionomies of Caatinga in Paraíba, Brazil. *Floresta* **49**, 287 (2019).

314. Souza, E. D. D. *et al.* Soil quality indicators after conversion of "murundu" fields into no-tillage cropping in the Brazilian Cerrado. *Pesqui. Agropecuária Bras* **54**, e00374 (2019).

315. Souza, E. D. *et al.* Soil quality indicators in a Rhodic Paleudult under long term tillage systems. *Soil Tillage Res* **139**, 28–36 (2014).

316. Souza, E. L. D. *et al.* Straw management effects on global warming potential and yield-scaled greenhouse gas emissions in a subtropical rice ecosystem. *Rev. Bras. Ciênc. Solo* **47**, e0220134 (2023).

317. Souza, L. H. C. *et al.* Soil carbon and nitrogen stocks and physical properties under no-till and conventional tillage cotton-based systems in the B razilian C errado. *Land Degrad. Dev* **29**, 3405–3412 (2018).

318. Steiner, F. *et al.* Organic carbon stock in soil affected by organic fertilization and cropping systems in southern Brazil. *Semina: Ciênc. Agrá* **33**, 2775-2788 (2012).

319. Teixeira, C. D. S. *et al.* Monocultures negatively influence ecosystem services provided by roots, plant litter and soil C stocks in subtropical riparian zones. *Environ. Dev. Sustain* **26**, 14729–14742 (2023).

320. Tenelli, S., De Oliveira Bordonal, R., Barbosa, L. C. & Carvalho, J. L. N. Can reduced tillage sustain sugarcane yield and soil carbon if straw is removed? *BioEnergy Res* **12**, 764–777 (2019).

321. Thomazini, A., Mendonça, E. S., Souza, J. L., Cardoso, I. M. & Garbin, M. L. Impact of organic no-till vegetables systems on soil organic matter in the Atlantic Forest biome. *Sci. Hortic* **182**, 145–155 (2015).

322. Tiecher, T. *et al.* Effect of 26-years of soil tillage systems and winter cover crops on C and N stocks in a Southern Brazilian Oxisol. *Rev. Bras. Ciênc. Solo* **44**, e0200029 (2020).

323. Tivet, F. *et al.* Aggregate C depletion by plowing and its restoration by diverse biomass-C inputs under no-till in sub-tropical and tropical regions of Brazil. *Soil Tillage Res* **126**, 203–218 (2013).

324. Tomaz, A. R. *et al.* Can natural undisturbed revegetation restores soil organic carbon to levels under native climax vegetation under tropical semiarid climate? *Land Degrad. Dev* **35**, 1971–1981 (2024).

325. Tonucci, R. G. *et al.* Agroforestry system improves soil carbon and nitrogen stocks in depth after land-use changes in the Brazilian semi-arid region. *Rev. Bras. Ciênc. Solo* **47**, e0220124 (2023).

326. Tornquist, C. G., Gassman, P. W., Mielniczuk, J., Giasson, E. & Campbell, T. Spatially explicit simulations of soil C dynamics in Southern Brazil: Integrating century and GIS with I Century. *Geoderma* **150**, 404–414 (2009).

327. Torres, J. L. R. *et al.* Soil physical attributes and organic matter accumulation under no-tillage systems in the Cerrado. *Soil Res* **57**, 712 (2019).

328. Tuchtenhagen, I. K., Lima, C. L. R. D., Bamberg, A. L., Guimarães, R. M. L. & Mansonia, P.-M. Visual Evaluation of the Soil Structure under Different Management Systems in Lowlands in Southern Brazil. *Rev. Bras. Ciênc. Solo* **42**, (2018).

329. Valadão, F. C. D. A., Maas, K. D. B., Weber, O. L. D. S., Valadão Júnior, D. D. & Silva, T. J. D. Variação nos atributos do solo em sistemas de manejo com adição de cama de frango. *Rev. Bras. Ciênc. Solo* **35**, 2073–2082 (2011).

330. Valladares, G. S. *et al.* Carbon and Nitrogen Stocks and Humic Fractions in Brazilian Organosols. *Rev. Bras. Ciênc. Solo* **40**, (2016).

331. Valpassos, M. A. R., Cavalcante, E. G. S., Cassiolato, A. M. R. & Alves, M. C. Effects of soil management systems on soil microbial activity, bulk density and chemical properties. *Pesqui. Agropecuária Bras* **36**, 1539–1545 (2001).

332. Veloso, M. G. et al. High carbon storage in a previously degraded subtropical soil under no-tillage with legume cover crops. *Agric. Ecosyst. Environ* **268**, 15–23 (2018).

333. Veloso, M. G., Cecagno, D. & Bayer, C. Legume cover crops under no-tillage favor organomineral association in microaggregates and soil C accumulation. *Soil Tillage Res* **190**, 139–146 (2019).

334. Vergutz, L. *et al.* Mudanças na matéria orgânica do solo causadas pelo tempo de adoção de um sistema agrossilvopastoril com eucalipto. *Rev. Bras. Ciênc. Solo* **34**, 43–57 (2010).

335. Vezzani, F. M. & Mielniczuk, J. Agregação e estoque de carbono em argissolo submetido a diferentes práticas de manejo agrícola. *Rev. Bras. Ciênc. Solo* **35**, 213–223 (2011).

336. Vicente, L. C., Gama-Rodrigues, E. F., Aleixo, S., Gama-Rodrigues, A. C. & Andrade, G. R. P. Chemical Composition of Organic Carbon in Aggregate Density Fractions Under Cacao Agroforestry Systems in South Bahia, Brazil. *J. Soil Sci. Plant Nutr* (2023).

337. Vizioli, B., Cavalieri-Polizeli, K. M. V. & Barth, G. Influence of ryegrass managements on the physical properties of a Haplohumox. *Pesqui. Agropecuária Bras* **53**, 952–960 (2018).

338. Vizioli, B., Cavalieri-Polizeli, K. M. V. & Barth, G. Silage yield, organic carbon content and physical attributes of a chiseled Ferralsol under an integrated crop-livestock system. *Rev. Bras. Ciênc. Agrár* **14**, 1–9 (2019).

339. Vogado, R.F. et al. Spatial variability of carbon and nitrogen stocks in integrated management systems and pasture in a Cerrado region. *Research, Society and Development* **9**, 1-21 (2020).

340. Wantzen, K. M. *et al.* Soil carbon stocks in stream-valley-ecosystems in the Brazilian Cerrado agroscape. *Agric. Ecosyst. Environ* **151**, 70–79 (2012).

341. Wendling, B. *et al.* Organic-Matter Lability and Carbon-Management Indexes in Agrosylvopasture System on Brazilian Savannah. *Commun. Soil Sci. Plant Anal* **39**, 1750–1772 (2008).

342. Wuaden, C. R., Nicoloso, R. S., Barros, E. C. & Grave, R. A. Early adoption of no-till mitigates soil organic carbon and nitrogen losses due to land use change. *Soil Tillage Res* **204**, 104728 (2020).

343. Xavier, F. A. D. S., Maia, S. M. F., De Oliveira, T. S. & De Sá Mendonça, E. Soil Organic Carbon and Nitrogen Stocks under Tropical Organic and Conventional Cropping Systems in Northeastern Brazil. *Commun. Soil Sci. Plant Anal* **40**, 2975–2994 (2009).

344. Zanatta, J. A., Vieira, F. C. B., Briedis, C., Dieckow, J. & Bayer, C. Carbon indices to assess quality of management systems in a Subtropical Acrisol. *Sci. Agric* **76**, 501–508 (2019).

345. Zeferino, L. B., Lustosa Filho, J. F., Dos Santos, A. C., Cerri, C. E. P. & De Oliveira, T. S. Soil carbon and nitrogen stocks following forest conversion to long-term pasture in Amazon rainforest-Cerrado transition environment. *CATENA* **231**, 107346 (2023).

346. Zinn, Yuri. L., Resck, D. V. S. & Da Silva, J. E. Soil organic carbon as affected by afforestation with Eucalyptus and Pinus in the Cerrado region of Brazil. *For. Ecol. Manag* **166**, 285–294 (2002).

347. Zortéa, T. *et al*. Grazing intensity and nitrogen fertilization timing to increase soil organic carbon stock and nitrogen in integrated crop-livestock systems. *Rev. Bras. Ciênc. Solo* **47**, e0230016 (2023).

348. Zotarelli, L. *et al*. Impact of Tillage and Crop Rotation on Aggregate-Associated Carbon in Two Oxisols. *Soil Sci. Soc. Am. J* **69**, 482–491 (2005).
