# Supplementary material for: Soil carbon debt from land use change in Brazil
Source: Nat Commun. 2026 Jan 26;17:1626. doi: 10.1038/s41467-026-68340-4 (PMC12905268; doi:10.1038/s41467-026-68340-4)
Supplement: Supplementary file 3 — Description of Additional Supplementary Files [file 41467_2026_68340_MOESM3_ESM.pdf]

Description of Additional Supplementary Files

File Name: Supplementary Data 1
Description: Database of soil carbon stocks in Brazilian biomes

File Name: Supplementary Data 2
Description: Database of pairwise comparisons between soil organic carbon stocks in native vegetation and agricultural areas

File Name: Supplementary Data 3
Description: Monocropping vs Integrated Agricultural Systems

File Name: Supplementary Data 4
Description: Monocropping vs. Cropping Rotation Intercropping

File Name: Supplementary Data 5
Description: Native Vegetation vs. Conventional Tillage

File Name: Supplementary Data 6
Description: Native Vegetation vs. cropping rotation intercropping

File Name: Supplementary Data 7
Description: Native Vegetation vs. Grassland

File Name: Supplementary Data 8
Description: Native Vegetation vs. Integrated Agricultural Systems

File Name: Supplementary Data 9
Description: Native Vegetation vs. Monocropping

File Name: Supplementary Data 10
Description: Native Vegetation vs. No-tillage

File Name: Supplementary Data 11
Description: Native Vegetation vs. Perennial crops
